# The transcriptional control of the VEGFA-VEGFR1 (FLT1) axis in alternatively polarized murine and human macrophages

**DOI:** 10.3389/fimmu.2023.1168635

**Published:** 2023-05-04

**Authors:** Apolka Domokos, Zsofia Varga, Karoly Jambrovics, Noemí Caballero-Sánchez, Eniko Szabo, Gergely Nagy, Beata Scholtz, Laszlo Halasz, Eszter Varadi, Krisztian P. Bene, Anett Mazlo, Attila Bacsi, Viktoria Jeney, Gabor J. Szebeni, Laszlo Nagy, Zsolt Czimmerer

**Affiliations:** ^1^ Department of Biochemistry and Molecular Biology, Faculty of Medicine, University of Debrecen, Debrecen, Hungary; ^2^ Doctoral School of Molecular Cell and Immune Biology, University of Debrecen, Debrecen, Hungary; ^3^ Institute of Genetics, Biological Research Centre, Eotvos Lorand Research Network, Szeged, Hungary; ^4^ Laboratory of Functional Genomics, Biological Research Centre Eotvos Lorand Research Network, Szeged, Hungary; ^5^ Departments of Medicine and Biological Chemistry, Johns Hopkins University School of Medicine, Institute for Fundamental Biomedical Research, Johns Hopkins All Children’s Hospital, St. Petersburg, FL, United States; ^6^ Doctoral School in Biology, University of Szeged, Szeged, Hungary; ^7^ Department of Immunology, Faculty of Medicine, University of Debrecen, Debrecen, Hungary; ^8^ ELKH-DE Allergology Research Group, Debrecen, Hungary; ^9^ MTA-DE Lendület Vascular Pathophysiology Research Group, Research Centre for Molecular Medicine, Faculty of Medicine, University of Debrecen, Debrecen, Hungary; ^10^ Department of Immunology, Albert Szent-Györgyi Medical School, Faculty of Science and Informatics, University of Szeged, Szeged, Hungary

**Keywords:** macrophage, IL-4, VEGFA, FLT1, hypoxia, transcriptional regulation, STAT6, EGR2

## Abstract

**Introduction:**

Macrophages significantly contribute to the regulation of vessel formation under physiological and pathological conditions. Although the angiogenesis-regulating role of alternatively polarized macrophages is quite controversial, a growing number of evidence shows that they can participate in the later phases of angiogenesis, including vessel sprouting and remodeling or regression. However, the epigenetic and transcriptional regulatory mechanisms controlling this angiogenesis-modulating program are not fully understood.

**Results:**

Here we show that IL-4 can coordinately regulate the VEGFA-VEGFR1 (FLT1) axis via simultaneously inhibiting the proangiogenic Vegfa and inducing the antiangiogenic Flt1 expression in murine bone marrow-derived macrophages, which leads to the attenuated proangiogenic activity of alternatively polarized macrophages. The IL-4-activated STAT6 and IL-4-STAT6 signaling pathway-induced EGR2 transcription factors play a direct role in the transcriptional regulation of the Vegfa-Flt1 axis. We demonstrated that this phenomenon is not restricted to the murine bone marrow-derived macrophages, but can also be observed in different murine tissue-resident macrophages ex vivo and parasites-elicited macrophages in vivo with minor cell type-specific differences. Furthermore, IL-4 exposure can modulate the hypoxic response of genes in both murine and human macrophages leading to a blunted Vegfa/VEGFA and synergistically induced Flt1/FLT1 expression.

**Discussion:**

Our findings establish that the IL-4-activated epigenetic and transcriptional program can determine angiogenesis-regulating properties in alternatively polarized macrophages under normoxic and hypoxic conditions.

## Introduction

Macrophages are found in almost all tissues as the heterogeneous and plastic cellular components of innate immunity. They play a multifaceted regulatory role under different physiological and pathological circumstances. In addition to the professional phagocytic and inflammation regulatory role, macrophages also participate in the maintenance of normal tissue homeostasis (1–3). Both the homeostatic and the antimicrobial activities are tightly determined in macrophages by their origin and molecular microenvironment. The tissue-resident macrophages can originate from embryonic progenitors forming self-renewal populations, such as microglial cells, Kupfer cells, and alveolar or large peritoneal macrophages. In contrast, intestinal macrophages can derive from embryonic progenitors and bone marrow monocytes (4–6). The bone marrow monocytes also play a critical role in the inflammatory response after pathogen infections or tissue injury (7, 8). The molecular microenvironment, including different immunomodulatory cytokines, lipids and metabolites, tightly determines the phenotypic features of macrophages and their response to different pathogen-derived and endogenous danger signals following infections or tissue injury. Two endpoints of the microenvironmental signals-induced functional macrophage polarization are the Th1-type cytokine interferon-gamma (IFNγ)-induced classical and Th2-type cytokines interleukin-4 (IL-4)- or IL-13-induced alternative macrophage polarization. These opposing macrophage polarization states can be characterized by completely distinct gene expression signatures and functional properties. The classically polarized macrophages contribute to the effective defense against bacterial infections. In contrast, alternative macrophage polarization is associated with a high tissue regenerative capacity and protective role against parasites infections. However, most pathological conditions have a complex molecular microenvironment resulting in various transient macrophage polarization forms with unique functional characteristics (9–12).

One of the important homeostatic functions of tissue-resident macrophages is monitoring the O_2_ level in the tissue microenvironment. In hypoxia, macrophages sense the low O_2_ level, which turns on hypoxia-specific transcriptional programs mediated by hypoxia-inducible factors (HIFs). The hypoxia-induced gene expression program incorporates the modified expression of several metabolism-related genes and the elevated production of proangiogenic factors, such as Vascular Endothelial Growth Factor A (VEGFA). The consequence of this specific response is endothelial cell proliferation and local angiogenesis that increases oxygenation in the given tissue (13, 14). However, hypoxia is not the sole proangiogenic trigger in macrophages. After tissue injury, the released endogenous and/or the pathogen-derived danger signals, as well as classical macrophage polarizing signal IFNγ induce highly pro-inflammatory phenotypes in both the tissue-resident and the recruited monocyte-derived macrophages associating with elevated VEGFA production and proangiogenic capacity (15–17). In the later stages of tissue regeneration and wound healing, the macrophage populations undergo a remarkable phenotypic shift from the pro-inflammatory characteristics to the anti-inflammatory or alternatively polarized phenotype in a minimum of three ways: (i.) direct conversion of the proinflammatory macrophages, (ii.) differentiation of newly recruited monocytes, or (iii.) local proliferation of macrophages at the site of injury (18–20). This phenotypic shift is associated with the changing angiogenesis-modulating role of the macrophages contributing to the regulation of the sprouting phase and the vessel remodeling and regression (17, 21).

Despite the important role attributed to alternatively polarized macrophages in tissue regeneration and hypoxic microenvironment-linked pathological processes, such as tumor development and progression (10, 12, 22–24), our knowledge about their angiogenesis-regulating function is incomplete and quite contradictory. It has been previously described that the alternatively polarized macrophages have high proangiogenic activity *in vitro* and *in vivo* using various angiogenesis assays (25, 26). However, other studies have not confirmed this phenomenon and raised the possibility that this macrophage type rather participates in the regulation of blood vessel regression during the resolution phase of wound repair (17, 21, 27). It has also been shown that macrophages play an essential role in the angiogenic response of the murine aortic ring to injury, but IL-4 and IL-13 stimulations can inhibit vessel formation (28). The putative antiangiogenic effect of IL-4 is also strengthened by the fact that IL-4 can induce an efficient VEGFA-neutralizing agent soluble VEGFR1 (Fms Related Receptor Tyrosine Kinase 1, FLT1) expression in macrophages, and the VEGFA-induced endothelial cell proliferation *in vitro* and laser-induced choroidal neovascularization *in vivo* are attenuated by IL-4-polarized macrophages in a sFLT1-dependent manner (29).

Through several steps, the Th2-type cytokine IL-4 can activate a specific transcriptional program in both murine and human macrophages regulating the phenotypic and functional characteristics of alternatively polarized macrophages. First, the IL-4-activated signal transducer and activator of transcription 6 (STAT6) can act as a transcription activator and repressor, directly regulating hundreds of genes, including many transcription factors, at the early phase of alternative macrophage polarization (30). After that, the IL-4-induced transcription factors, such as Krüppel-like Factor 4 (KLF4), Interferon regulatory factor 4 (IRF4), c-Myc protooncoprotein (cMYC), Peroxisome proliferator-activated receptor gamma (PPARγ) and Early growth response protein 2 (EGR2) are responsible for the epigenetic and transcriptional bases of the late phase of alternative macrophage polarization and the transcriptional memory (31–36). Despite the available information about the angiogenesis regulating role of distinct macrophage polarization states, the epigenetic and transcriptional mechanisms controlling the expression of FLT1 and its proangiogenic ligands in the alternatively polarized macrophages are not completely understood.

Here we uncovered how STAT6 and EGR2 transcription factors directly regulate the elevated *Flt1* and transiently repressed *Vegfa* expression at the transcriptional level during alternative macrophage polarization in murine bone marrow-derived macrophages (BMDMs). We found that the IL-4-STAT6 signaling pathway could also repress the hypoxia-induced *Vegfa* production in parallel with the synergistic induction of *Flt1* expression. The regulatory effects of IL-4 on the *Flt1-Vegfa* axis were not restricted to the murine BMDMs; this phenomenon was also observed in different tissue-resident macrophage subsets *ex vivo* and nematode-elicited macrophages *in vivo*. Finally, partial evolutionary conservation was found in the IL-4-dependent regulation of VEGFA/*Vegfa* and FLT1/*Flt1* expression between human and murine macrophages under normoxic and hypoxic conditions.

## Materials and methods

### Mice

Female and male breeder mice for C57BL/6 strain were used and bred under specific-pathogen-free (SPF) conditions. *Egr2^fl/fl^ Lyz2-cre and Stat6^−/−^* animals were kept on the C57BL/6 genetic background. The *Egr2^fl/fl^* animals were a generous gift from Patrick Charnay’s laboratory. We crossed these mice with lysozyme M-Cre (*Lyz2-cre*)+ animals to establish the conditional EGR2-deficient strain (*Egr2^fl/fl^ Lyz2-cre*). These mice were backcrossed to the C57BL/6J strain for eight generations. As controls, we used *Egr2^+/+^ Lyz2-cre* littermates. Full-body *Stat6^-/-^* mice are available through Jackson Laboratory. Full-body knockout animals were maintained by breeding STAT6-deficient male and female mice. Wild-type C57BL/6 mice were used as controls. Animals were handled according to the regulatory standards of the animal facilities of the University of Debrecen. 8 to 12-week-old healthy male mice were used for all our experiments. Animal studies were approved by the Animal Care and Protection Committee at the University of Debrecen (16/2019/DE MAB).

### Bone marrow-derived macrophage differentiation and treatment conditions

Isolation and differentiation were completed as described earlier (37). For the differentiation of bone marrow-derived cells, the *humerus*, *femur*, and *tibia* of C57BL/6 wild-type, *Stat6^-/-^* and *Egr2^fl/fl^Lyz2-cre* mice were processed.

Bone marrow-derived cells were differentiated for 6 days in DMEM (Sigma; D5671) containing L929 supernatant (high glucose DMEM with 1% penicillin/streptomycin/amphotericin b, 15% FBS, 20% L929 supernatant). For further treatment of macrophages, L929 supernatant-free DMEM was implemented (high glucose DMEM with 1% penicillin/streptomycin/amphotericin b, 15% FBS, 0.16% L-glutamine). Cultures were treated with a final concentration of 20 ng/ml IL-4 (Peprotech; 214–14) and 1 µM STAT6 phosphorylation inhibitor AS1517499 (Sigma; SML1906) for the indicated period. Hypoxic conditions were obtained by a continuous flow (0.1 L/minute) of a gas mixture (1% O_2_, 5% CO_2_, 94% N_2_).

### Bone marrow-derived mesenchymal stem cells culture

Bone marrow-derived mesenchymal stem cells (MSCs) from C57BL/6 mice were isolated and characterized as previously described (38, 39). The MSCs were cultured in DMEM/Ham’s F-12 medium (Capricorn Scientific, DMEM-12-A) supplemented with 10% fetal bovine serum (FBS) (Gibco®, A3840401), 100 U/mL penicillin-streptomycin (Gibco®, Thermo Fischer Scientific, 15140122) and 2 mM L-glutamine (Gibco®, Thermo Fisher Scientific, 25030081) in a humidified incubator with 5% CO_2_ at 37°C.

Mouse heart endothelioma cells, H5V (kindly provided by Csaba Vizler from Biological Research Centre, Szeged, Hungary) were cultured in DMEM/Ham’s F-12 (Capricorn Scientific, DMEM-12-A) supplemented with 10% FBS (EuroClone, ECS0180L), 100 U/mL penicillin-streptomycin (Gibco®, Thermo Fischer Scientific, 15140122) and 2 mM L-glutamine (Gibco®, Thermo Fisher Scientific, 25030081) in a humidified incubator with 5% CO_2_ at 37°C.

### Bronchoalveolar lavage isolation

For bronchoalveolar lavage (BAL) collection, the trachea of euthanized mice were cannulated. Lavage was performed with 2 aliquots of 0.7 ml of ice-cold PBS (pH 7.3). The BAL cells were centrifuged (800 rcf, 10 minutes (min), 4°C). Supernatants were stored at -80°C until further analysis. Collected BAL cells after red blood cell lysis with ACK lysis buffer (room temperature (RT), 2 min) were washed with MACS buffer (800 rcf, 10 min, 4°C) and then were used for flow cytometry and cell sorting.

### Peritoneal lavage isolation

The mice were euthanized, and the peritoneal cavity was washed with 8 ml PBS (pH 7.3). The obtained cells were filtered through a 100 µm cell strainer. Following centrifugation (350xg, 5 min, 4°C) pellets were suspended with 2 ml ACK lysis buffer for 2 min for red blood cell lysis at RT. Cell suspensions were washed with 10 ml MACS buffer and cells were used for flow cytometry and cell sorting.

### Alveolar and peritoneal macrophage isolation, flow cytometry, and cell sorting

BAL-derived cells were labeled for anti-mouse CD11c-phycoerythrin (PE, clone HL3, BD Biosciences) and anti-mouse F4/80-allophycocyanin (APC, clone BM8, BioLegend) antibodies. Peritoneum-derived cells were labeled for anti-mouse F4/80-APC and anti-mouse CD11b-PE- Cyanine7 (PE-Cy7, clone M1/70, eBioscience).

The FcR Blocking Reagent (Miltenyi Biotec) was used to increase specificity by preventing non-speciic binding of antibody conjugates. To exclude dead cells, eBioscience^™^ Fixable Viability Dye eFluor^™^ 506 (Thermo Fischer Scientific) was applied based on the manufacturer’s instructions.

The CD11c-F4/80 double-positive alveolar macrophages and F4/80hi-CD11bhi large peritoneal macrophages were sorted.

The flow cytometry analysis and cell sorting were performed by BD FACSAria^™^ III (BD Biosciences) using BD FACSDiva Software 6.0 (BD Biosciences). Flow cytometry data analysis was performed with FlowJo v10.8 (BD Biosciences).

### *Ex vivo* alveolar and peritoneal macrophage polarization and activation

Isolated cells were cultured at 24-well plates in a 0.5 mL RPMI medium containing 5% penicillin/streptomycin/amphotericin b and 10% FBS. After 1 hour (h) of attachment, 20 ng/ml IL-4 was added for 24 h.

### Human monocyte isolation and differentiation

Peripheral blood was collected from healthy volunteers from the Regional Blood Center of the Hungarian National Blood Transfusion Service (Debrecen, Hungary) with the approval of the Regional Institutional Research Ethics Committee of the University of Debrecen.

Human monocytes were separated as it was published previously (40). After density gradient centrifugation with Ficoll Plaque Plus (Amersham Biosciences; GE17-1440-02) the peripheral blood mononuclear cells (PBMCs) were isolated from the buffy coats. Positive selection of human monocytes from PBMCs was carried out using anti-CD14-conjugated MicroBeads (Miltenyi Biotec; 130-050-201) in accordance with the manufacturer’s protocol.

Isolated monocytes were suspended in RPMI 1640 (Sigma; D5671) media supplemented with 10% FBS and 5% penicillin/streptomycin/amphotericin b. In a 6-well cell culture plate 2x10^6^ cells/ml were plated and placed in a humidified incubator at 37°C atmosphere containing 5% CO_2_ for 16 h before treatment. Adherent, monocyte-derived differentiating macrophages were treated with 20 ng/ml IL-4 (Peprotech; 200–04) under normoxic and hypoxic conditions with for the indicated period.

### Enzyme-linked immunosorbent assay

Cell culture supernatants were collected at different time points. After centrifugation (1000 rcf, 10 min, 4°C) supernatants were stored at -20°C until further analysis.

Protein levels of VEGFA and FLT1 were determined using Mouse VEGF DuoSet ELISA Kit (R&D Systems; DY493) and Mouse VEGFR1/FLT-1 Quantikine ELISA Kit (R&D Systems; MVR100), in accordance with the instructions provided by the manufacturer. The plates were read using the BIO-TEK Synergy HT Multi-Detection Microplate Reader.

### Immunoblot

Treated cells were pelleted and lysed in lysis buffer (50 mM TRIS, 1mM EDTA, 0.1% mercaptoethanol, 0.5% Triton X-100, and 1 mM PMSF) containing a protease inhibitor and phosphatase inhibitor cocktail (Sigma-Aldrich) with a 1:100 dilution ratio and homogenized with 5–7 strokes with a sonicator (Branson Sonifer, 450) at 40% cycle intensity. The lysed samples were centrifuged at 18213 g at 4°C for 15 minutes, then the supernatant was used for protein measurements with the Bradford assay at a wavelength of 595 nm (Synergy Multi-Mode Microplate Reader). Each sample was measured in triplicate. The protein samples were diluted to 2 mg/mL, mixed with equal volumes of 2×SDS denaturation buffer (0.125 M Tris-HCl, pH 6.8, containing 4% SDS, 20% glycerol, 10% mercaptoethanol, and 0.02% bromophenol blue), and incubated at 99°C for 10 minutes. Depending on their molecular weights, the proteins were separated on 8–10% SDS polyacrylamide gels and then blotted onto a PVDF membrane (Merck-Millipore), using a semi-dry blotting method. The membranes were blocked with 5% nonfat dry milk/5% BSA in Tris-buffered saline and Tween 20 (TTBS) for 1 h at RT. Primary antibodies were diluted in 0.5% milk/5% BSA in TTBS at a dilution ratio of 1:1000–1:5000 and incubated overnight at 4°C. Membranes were washed three times with TTBS for 15 min at RT, incubated with horseradish peroxidase-labeled, affinity-purified secondary antibodies (Advansta) at 1:10000–1:20000 dilution for 1 h at RT, and then washed three times with TTBS for 15 min at RT. The targeted protein bands were visualized using an ECL Kit (Advansta). The protein bands were quantified using ImageJ software, version 1.09.

### *In vitro* capillary formation assay

Co-culture of MSCs and H5Vs was initiated on a 24-well plate at a cell density of 2.5 × 10^4^ H5V and 1.5 × 10^4^ MSC cells/well. The experiments were carried out in triplicates. L929 supernatant-free DMEM or the supernatants of BMDMs generated and culture medium of MSCs were added in 1:4 ratio in a final volume of 400 mL or treated with 4 ng/mL IL-4. After 3 days of co-culture, cells were fixed with 2% paraformaldehyde. Tube formation was evaluated as follows: Images were taken of 8 randomly selected non-overlapping areas per well using Olympus Cell-R fluorescence microscope (Olympus Holding Europa GmbH) or Visitron VisiScope Spinning Disc Confocal microscope (Visitron Systems GmbH) with UPlanSApo 4x/0.16 and 4x/0.13 objectives, respectively. The lengths of pre-vascular structures were measured with Fiji/ImageJ Software, and the total length of the structure network per area was calculated.

### Real-time quantitative PCR for enhancer RNA and messenger RNA detection

RNA was isolated with TRIzol reagent (Ambion; 15596018). High-Capacity cDNA Reverse Transcription Kit (Applied Biosystems; 4368813) was used for the reverse transcription of RNA into cDNA according to the manufacturer’s protocol. Transcript quantification was performed by RT-qPCR reactions using LightCycler 480 SYBR Green I Master mix (Roche; 4887352001). Transcript expressions were normalized to the β-actin (ACTB) housekeeping gene. Primers are available upon request.

### Gene expression and transcript variant analyses from publicly available RNA-sequencing datasets

Publicly available RNA-seq datasets can be found in GEO and SRA repositories. (IL-4 treated BMDM GEO accession: GSE106706. Tissue-resident macrophages dataset GEO accession: GSE63341. Datasets of *Brugia malayi* implantation model SRA accession: ERP001255.) RNA-seq datasets were analyzed on Galaxy web platform (41). The sequencing quality was evaluated by FastQC Read Quality reports software (Galaxy Version 0.73+galaxy0) and paired-end reads were mapped to the mouse reference genome (mm10) using HISAT2 (Galaxy Version 2.2.1+galaxy1) (42). Read counts were determined with featurecounts package (Galaxy Version 2.0.1+galaxy2) (43) and the normalized gene expressions were calculated by DESeq2 (Galaxy Version 2.11.40.7+galaxy2) (44).

### Analysis of publicly available assay for transposase-accessible chromatin using sequencing dataset

Raw chromatin accessibility data from tissue-resident macrophages were downloaded from GEO under GSE63338 accession number. Alveolar macrophage data from *Egr2^+/+^* and *Egr2^fl/fl^* were downloaded from GEO under GSE181087 accession number. Sequencing quality was evaluated by FastQC software. Read alignment and filtering were carried out using our command line pipeline (45). Briefly, reads were mapped to the mouse reference genome (*mm10*) using the default parameters of BWA *MEM* aligner (46). Low mapping quality reads (MAPQ < 10), reads mapping to ENCODE mouse blacklisted regions (47) and duplicated reads were discarded from the downstream analyses, using bedtools intersectBed (48) and samtools rmdup (49). MACS2 (50) was used to call peaks at 5% false discovery rate (FDR). Coverage profiles represent Reads Per Kilobase Million (RPKM) values, calculated using deeptools2 bamCoverage (51) and visualized in IGV (52).

### Analysis of publicly available chromatin immunoprecipitation followed by sequencing and cleavage under targets and release using nuclease sequencing datasets

ChIP-seq data (H3K4me2 and H3K27Ac) from tissue-resident macrophages were downloaded from GEO under GSE63339 accession number. STAT6, EGR2, H3K27Ac and RNAPII-pS2 ChIP-seq data were downloaded from GEO under GSE151015 accession number. Alveolar macrophage data (H3K4m3; CUT&RUN sequencing) from *Egr2^+/+^* and *Egr2^fl/fl^* were downloaded from GEO under GSE181087 accession number. Human and mouse PU.1 and STAT6 (0- 30 min IL-4 treatment) ChIP-seq data were downloaded from GEO under GSE1008899 accession number. Sequencing quality was evaluated by FastQC software. Read alignment and filtering were carried out using our command line pipeline (45). Briefly, reads were mapped to the mouse reference genome (*mm10*) using the default parameters of BWA MEM aligner (46). Low mapping quality reads (MAPQ < 10), reads mapping to ENCODE mouse blacklisted regions (47) and duplicated reads were discarded from the downstream analyses, using bedtools intersectBed (48) and samtools rmdup (49). MACS2 (50) was used to call peaks at 5% false discovery rate (FDR). Coverage profiles represent Reads Per Kilobase Million (RPKM) values, calculated using deeptools2 bamCoverage (51) and visualized in IGV (52).

### Analysis of publicly available single-cell RNA-sequencing dataset

Data from naïve (GSM6040532 and GSM6040533) and *Trypanosoma brucei-*infected (45 days after the injection) mice-derived brain samples (GSM6040536 and GSM6040537) were collected from the public GEO database. The gene expression matrixes were filtered by sample, selecting those cells with at least 100 genes or higher, less than 20% of mtRNA and removing doublets and multiplex (around 7% of cells). The analysis was performed using Seurat v4.3.0. After gene expression normalization and scaling to remove variance between cells, the cells were clustered using the Seurat workflow based on dimensionality reduction by a Principal Component Analysis (PCA). The first 5 PCs were used to identify the different clusters in the dataset and to visualize these clusters in a t-Distributed Stochastic Neighbor Embedding (tSNE) plot. Cells were annotated using SingleR package and ImmGen database and curated manually. From the dataset, monocytes, macrophages, and microglia were selected to proceed with the analysis, re-clustering them using the first 4 PCs. Replicates were integrated using harmony, and the representation of the normalized expression was performed using the function VlnPlot and the package Nebulosa.

### Transcription factor binding motif and conservation analyses

The *Vegfa* and *Flt1* murine (*mm10*) genomic loci were converted to human (*hg19*) loci in 100-bp resolution using liftOver (UCSC). Human genomic segments closer than 100 bp to each other were united and marked alternately with green, burgundy, and blue colors using command line tools. Mouse genomic segments orthologous to these were marked with the same color. “phastCons100way” per nucleotide conservation scores were downloaded from the UCSC Table Browser and for visualization in the IGV genome browser, they were converted to bedgraph format using command line tools. General STAT (TTCYNRGAA) and STAT6-specific (TTCYNNRGAA) sequences were mapped by annotatePeaks.pl (HOMER).

### Statistical analysis

The error bars represent the standard deviation (SD). The two-tailed Student’s test-test was used to evaluate the significance of differences between two groups. Quantification and alignments of NGS analysis. Results with p-values of less than 0.05 were considered statistically significant (#p<0.1, *p <0.05, **p <0.01, ***p <0.001 and ns = not significant change).

## Results

### Coordinated but opposite regulation of *Flt1* and *Vegfa* mRNA expression in alternatively polarized murine BMDMs

**Figure 1:**
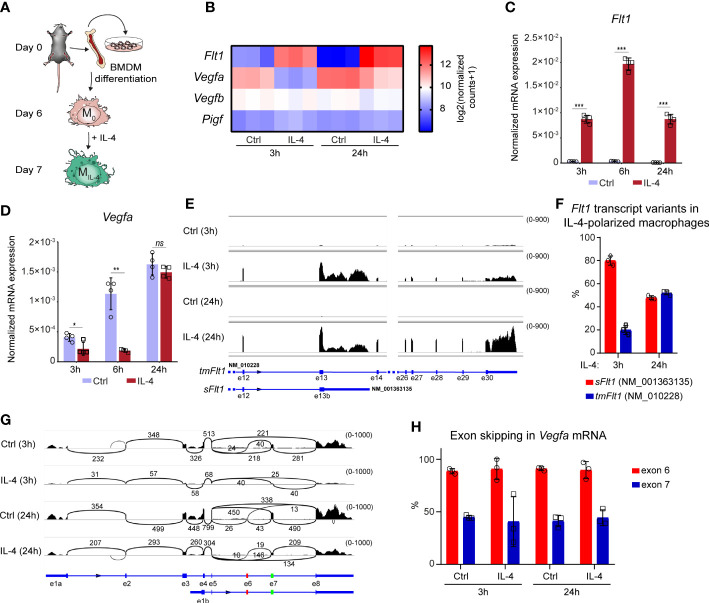
Opposing regulation of *Flt1* and *Vegfa* mRNA expression in the alternatively polarized murine BMDMs. **(A)** Schematic representation of the mouse bone marrow isolation, BMDM differentiation and polarization by IL-4. **(B)** Heatmap showing the mRNA expression levels of *Flt1* and its potential ligands *Vegfa*, *Vegfb* and *Pigf* in non-polarized and alternatively polarized wild-type murine BMDMs at 3- and 24-hours following IL-4 stimulation (n=3). **(C)** RT-qPCR measurements of *Flt1* mRNA expression from IL-4-polarized and non-polarized wild-type BMDMs at three indicated time points. Bar graphs present the mean ± SD of four biological replicates. **(D)** RT-qPCR measurements of *Vegfa* mRNA expression from IL-4-polarized and non-polarized wild-type BMDMs at three indicated time points. Bar graphs present the mean ± SD of four biological replicates. **(E)** The shortened soluble (*sFlt1*, NM_001363135) and full-length, transmembrane (*tmFlt1*, NM_010228) *Flt1* transcript variants in non-polarized and alternatively polarized wild-type murine BMDMs at 3- and 24-hours following IL-4 stimulation visualized by the Integrative Genomics Viewer. The image was derived from merged bam files of 3 replicates for each condition, with the range of coverage shown to the upper left of each track, between parentheses. **(F)** The ratio of the soluble *Ftl1* (*sFlt1*, NM_001363135) and the transmembrane (*tmFlt1*, NM_010228) *Flt1* transcript variants in non-polarized and alternatively polarized wild-type murine BMDMs at 3- and 24-hours following IL-4 stimulation. Bar graphs present the mean ± SD of three biological replicates. **(G)** Sashimi plots showing the transcript isoforms and alternative splicing of *Vegfa*, visualized by the Integrative Genomics Viewer. Arcs are between exons connected by splicing, with the number of junctional reads indicated above. The image was derived from merged bam files of 3 replicates for each condition, with the range of coverage shown to the upper left of each track, between parentheses. Exons 6 and 7, affected by exon skipping, are highlighted in red and green on the gene model below. The short transcript isoform with alternative exon 1b represents NM_001110267.1 (major variant, no exon 6), NM_001110268.1 (no exon 6 or 7), and NM_001110266.1 (no exon skipping). **(H)** The ratio of *Vegfa* exon 6 and 7 skipping in non-polarized and alternatively polarized wild-type murine BMDMs at 3- and 24-hours following IL-4 stimulation. Bar graphs present the mean ± SD of three biological replicates. *p <0.05, **p <0.01, ***p <0.001, ns, not significant change.

To investigate whether IL-4 can directly modulate the angiogenesis-regulating role of macrophages, we started to study the production of antiangiogenic factor FLT1 and its proangiogenic ligands, including VEGFA, VEGFB, and PIGF, in non-polarized and alternatively polarized murine BMDMs (the experimental scheme is shown in **Figure 1A**). First, we determined their steady-state mRNA expression levels during alternative macrophage polarization at two different time points after IL-4 exposure using our previously published RNA-seq data set (30). Similarly to the well-known alternative macrophage polarization markers - *Arg1*, *Retnla*, and *Chil3 -* the *Flt1* mRNA expression was induced at 3 and 24 hours following the IL-4 stimulation (**Supplementary Figure 1** and **Figure 1B**). Among the potential FLT1 ligands, *Vegfb* and *Pigf* genes were expressed in macrophages, but their mRNA expression levels were not affected significantly (**Figure 1B**). In contrast, the *Vegfa* mRNA expression was already reduced following 3 hours of IL-4 stimulation, but this attenuating effect was less detectable after 24 hours (**Figure 1B**). To validate the RNA-seq data and further investigate the dynamics of the IL-4-regulated *Vegfa* and *Flt1* expression, we measured their mRNA levels at 3, 6, and 24 hours following IL-4 stimulation in murine BMDMs by RT-qPCR. As shown in **Figure 1C**, the IL-4-induced *Flt1* mRNA expression was detected at each examined time point, but the highest induction level was observed 6 hours after IL-4 stimulation (**Figure 1C**). In contrast, significant IL-4-repressed *Vegfa* mRNA expression was detected only at the early (3 and 6 hours) time points (**Figure 1D**).

*Flt1* and *Vegfa* gene expression are also regulated in various cell types by alternative splicing (53, 54); therefore, we aimed to map the expression of their different transcript variants in the non-polarized and alternatively polarized BMDMs in the above-used RNA-seq dataset. We could detect two *Flt1* transcript variants, including shortened soluble (*sFlt1*, NM_001363135) and full-length transmembrane (*tmFlt1*, NM_010228) isoforms in the IL-4 polarized macrophages, but their ratio proved to be different at the two examined time points following IL-4 stimulation (**Figures 1E, F**). While the shortened *sFlt1* transcript variant was dominant (80%) in the early phase (3h), their proportion was almost the same in the late phase (24h) of alternative macrophage polarization (**Figure 1F**). Additionally, we could detect the long and short isoforms of *Vegfa* (the short isoform lacking exons 1-3, with an alternative 1^st^ exon), and exon skipping affecting exon 6 and/or 7 in different ratios in the murine macrophages. The ratio of long/short *Vegfa* isoforms increased minimally after IL4 stimulation, but the exon skipping pattern remained the same (**Figures 1G, H**).

Taken together, our findings indicate that IL-4 counter-regulates *Flt1* and *Vegfa* mRNA expression, raising the possibility that IL-4 modifies the angiogenic capacity of murine macrophages.

### The IL-4-induced sFLT1 secretion is associated with diminished VEGFA production and attenuated proangiogenic capacity in murine alternatively polarized macrophages

**Figure 2:**
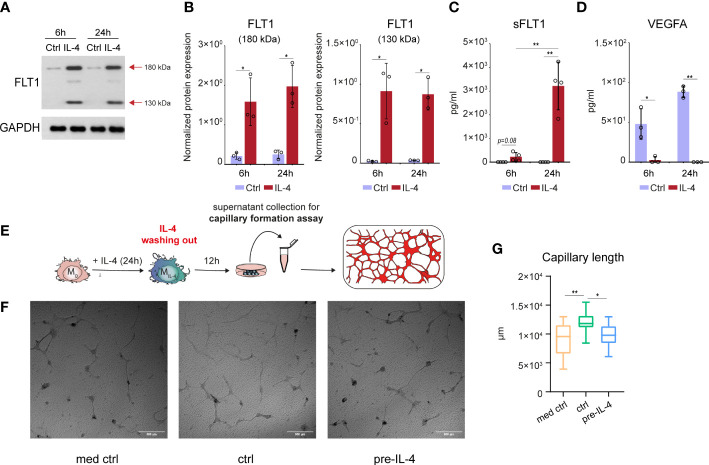
IL-4-induced soluble FLT1 production is associated with decreased VEGFA secretion and reduced proangiogenic capacity in alternatively polarized murine BMDMs. **(A)** Immunoblot of FLT1 expression in non-polarized and IL-4-exposed wild-type murine BMDMs at the indicated time points. One representative blot is shown from three independent experiments. GAPDH protein expression serves as a loading control. **(B)** Immunoblot densitometry analysis of two different isoforms of FLT1 protein in IL-4-polarized and non-polarized wild-type murine BMDMs at the indicated time points. Data represent the mean and SD of three individual animals. **(C)** ELISA measurements on the soluble FLT1 protein in culture supernatants derived from IL-4-polarized and non-polarized murine BMDMs at the indicated time points. Bar graphs present the mean ± SD of four biological replicates. **(D)** ELISA measurements on the VEGFA protein in culture supernatants derived from IL-4-polarized and non-polarized murine BMDMs at the indicated time points. Bar graphs present the mean ± SD of four biological replicates. **(E)** Schematic representation of the supernatant collection from the alternatively polarized and non-polarized murine BMDMs and *in vitro* capillary formation assay. **(F)** Capillary formation in murine MSC and H5V heart capillary endothelial cell coculture-based *in vitro* angiogenesis assay exposed to supernatants from non-polarized and IL-4 pre-conditioned murine BMDMs. **(G)** Box plot representation of capillary length (µm) in murine MSC and H5V coculture exposed to supernatants from non-polarized and IL-4 pre-conditioned murine BMDMs (n=15). *p <0.05, **p <0.01.

To examine the potential IL-4-reduced proangiogenic ability of macrophages, first we determined the FLT1 protein production by non-polarized and alternatively polarized murine BMDMs using the immunoblot method. As shown in **Figures 2A, B**, our immunoblot analysis detected a robust induction of the ~180 kDa molecular weight isoform of FLT1 protein in the alternatively polarized BMDMs at both examined time points. Besides, the smaller~130 kDa molecular weight FLT1 isoform was also identified at 6 hours after IL-4 stimulation, and its expression was enhanced at 24 hours by IL-4. To investigate whether alternatively polarized macrophages can secrete FLT1, we measured soluble FLT1 (sFLT1) content in the cell culture media derived from untreated and IL-4-stimulated murine BMDMs by ELISA. Although we already could detect significantly elevated sFLT1 levels in the cell culture media 6 hours after IL-4 stimulation, the most robust sFLT1 production was observed 24 hours following the IL-4 treatment (**Figure 2C**). Next, to determine the direct and indirect sFLT1-mediated repressive effects of IL-4 on the available biologically active VEGFA protein level, we measured the secreted VEGFA content in the cell culture supernatants using the ELISA method. The almost complete reduction of available VEGFA protein level was observed in the supernatants derived from the alternatively polarized macrophages at both indicated time points following the IL-4 exposure (**Figure 2D**) indicating that the regulatory role of IL-4 is not restricted to the transcriptional control of *Vegfa* expression in murine macrophages.

In order to demonstrate the functional consequences of the IL-4-regulated FLT1-VEGFA axis in murine BMDMs, we studied the proangiogenic capacity of non-polarized and IL-4-polarized BMDMs-derived supernatants using an *in vitro* capillary formation assay based on mesenchymal stromal cell and H5V mouse heart capillary endothelial cell co-culture. Since the direct antiangiogenic effect of IL-4 on endothelial cells is described previously (55), we wanted to test whether the IL-4 content of the alternative polarized macrophage-derived media can directly modulate the capillary formation in the applied *in vitro* assay. As shown in **Supplementary Figures 2A, B**, the exogenously added IL-4 alone had a significant antiangiogenic effect in our experimental system. To exclude the direct capillary formation regulating role of IL-4, we removed the cell culture media from the macrophages following 24 hours of IL-4 exposure, and we further cultured them in IL-4-free media for 12 hours before the collection of the supernatants (the experimental scheme is shown in **Figure 2E**). We found that the VEGFA production by IL-4-primed BMDMs was remarkably reduced at the end of the 12-hour resting period by IL-4-primed BMDMs compared to the unstimulated counterparts (**Supplementary Figure 2C**). In the *in vitro* capillary formation assay, the non-polarized BMDM-derived supernatant significantly enhanced capillary formation, but this proangiogenic effect was completely absent in the case of the IL-4-primed macrophage-derived supernatant (**Figures 2F, G**).

Taken together, these findings indicate that IL-4 also oppositely regulates FLT1 and VEGFA at the biologically available secreted protein level in murine BMDMs, in line with the reduced proangiogenic capacity of macrophages.

### STAT6 transcription factor directly contributes to the IL-4-mediated regulation of *Flt1* and *Vegfa* expression

**Figure 3:**
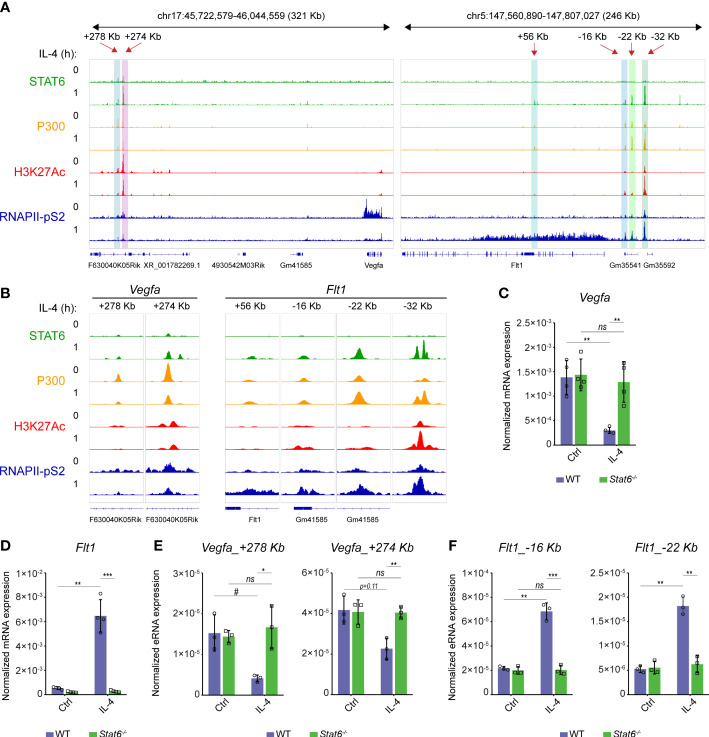
The direct STAT6 transcription factor-mediated repression of *Vegfa* and induction of *Flt1* transcription at the early phase of alternative macrophage polarization in murine BMDMs. **(A)** STAT6, P300, H3K27Ac, RNAPII-pS2-specific ChIP-seq signals in IL-4-polarized and non-polarized murine BMDMs at the *Vegfa* and *Flt1* loci visualized by the Integrative Genomics Viewer. **(B)** STAT6, P300, H3K27Ac, RNAPII-pS2-specific ChIP-seq signals in IL-4-polarized and non-polarized murine BMDMs at the STAT6-bound *Vegfa* and *Flt1*-associated distal regulatory regions visualized by the Integrative Genomics Viewer. **(C)** RT-qPCR measurements of *Vegfa* mRNA expression from IL-4-polarized and non-polarized wild-type (WT) and *Stat6^-/-^* murine BMDMs at 3 hours following IL-4 stimulation. Bar graphs present the mean ± SD of four biological replicates. **(D)** RT-qPCR measurements of *Flt1* mRNA expression from IL-4-polarized and non-polarized wild-type (WT) and *Stat6^-/-^* murine BMDMs at 3 hours following IL-4 stimulation. Bar graphs present the mean ± SD of four biological replicates. **(E)** RT-qPCR measurements of eRNA expression at *Vegfa*_+278kb and *Vegfa*_+274Kb enhancers from IL-4-polarized and non-polarized wild-type (WT) and *Stat6^-/-^* murine BMDMs at 3 hours following IL-4 stimulation. Bar graphs present the mean ± SD of three biological replicates. **(F)** RT-qPCR measurements of eRNA expression at *Flt1*_-16Kb and *Flt1*_-22Kb enhancers from IL-4-polarized and non-polarized wild-type (WT) and *Stat6^-/-^* murine BMDMs at 3 hours following IL-4 stimulation. Bar graphs present the mean ± SD of three biological replicates. #p<0.1, *p <0.05, **p <0.01, ***p <0.001, ns, not significant change.

To better understand the opposing regulation of *Flt1* and *Vegfa* mRNA expression at the early time points of alternative macrophage polarization, we reanalyzed our previously published RNA Polymerase II (RNAPII), coactivator P300, active histone mark H3K27Ac, and STAT6-specific ChIP-seq datasets from both IL-4-stimulated and unstimulated murine BMDMs (30, 36). The RNAPII binding was reduced at the gene body of *Vegfa* while it was dramatically induced at the *Flt1*-coding genomic region following 1-hour IL-4 stimulation, indicating that IL-4 regulates their expression at the transcription level (**Figure 3A**). Besides, IL-4-activated STAT6 transcription factor binding was observed at many distal regulatory regions in both genomic loci (**Figures 3A, B**). However, the effect of the short-term IL-4 exposure on RNAPII, P300 binding and H3K27Ac pattern showed remarkable differences between the *Flt1* and *Vegfa*-associated enhancers. At the previously described distant enhancers assigned to *Vegfa*, including *Vegfa*_+278Kb and *Vegfa*_+274Kb (37), the STAT6 binding was associated with attenuated P300 and RNAPII binding, as well as H3K27Ac following 1-hour IL-4 stimulation (**Figures 3A, B**). In contrast, short-term IL-4 exposure could induce the P300 and RNAPII binding, as well as H3K27Ac, at many distal regulatory regions in the *Flt1* locus (**Figures 3A, B**).

To confirm the bidirectional regulatory role of the IL-4-activated STAT6 transcription factor in the transcriptional control of the *Flt1*-*Vegfa* axis, we measured the steady-state *Flt1* and *Vegfa* mRNA expression levels and the enhancer RNA (eRNA) expression at 2 selected enhancers from both genomic loci in wild-type and STAT6-deficient murine BMDMs using RT-qPCR. IL-4-induced *Flt1* and IL-4-repressed *Vegfa* expression proved to be completely STAT6-dependent at 3 hours after the IL-4 stimulation (**Figures 3C, D**). Similarly to the regulation of the steady-state *Flt1* and *Vegfa* mRNA expression, IL-4-dependent changes in the eRNA expression at the selected enhancers, including *Vegfa*_+278Kb, *Vegfa*_+274Kb, *Flt1*_-16Kb, and *Flt1*_-22Kb, also showed STAT6 dependency (**Figures 3E, F**).

Overall, these findings suggest that the IL-4-STAT6 signaling pathway directly but oppositely controls the *Vegfa* and *Flt1* expression at the transcriptional level in the early phase of alternative macrophage polarization in murine BMDMs.

### EGR2 transcription factor plays an important role in the regulation of *Flt1* and *Vegfa* expression at the late phase of alternative macrophage polarization

**Figure 4:**
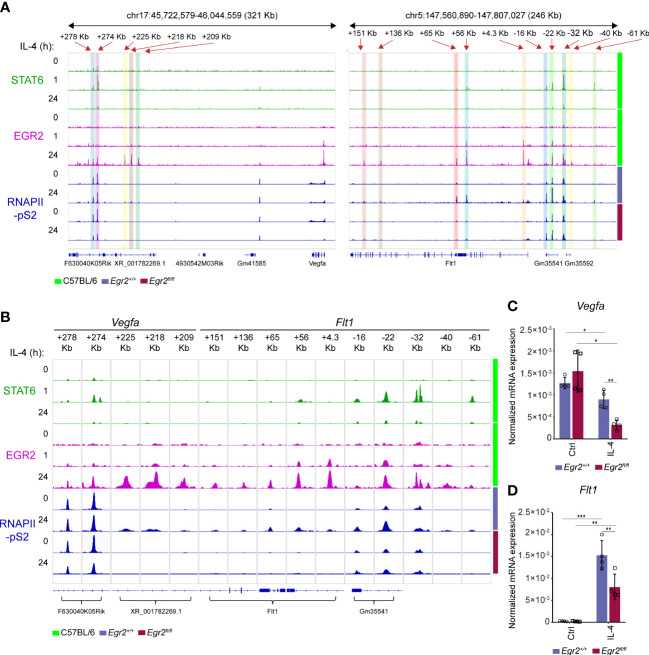
IL-4 mediated *Vegfa* repression and *Flt1* induction are partially EGR2 transcription factor-dependent at the late phase of alternative macrophage polarization. **(A)** STAT6 and EGR2-specific ChIP-seq signals in wild-type murine BMDMs after 0-, 1- and 24-hours IL-4 stimulation, as well as RNAPII-pS2-specific ChIP-seq signals in non-polarized and IL-4-polarized wild-type (*Egr2^+/+^*) and EGR2-deficient (*Egr2^fl/fl^*) murine BMDMs at the *Vegfa* and *Flt1* loci visualized by the Integrative Genomics Viewer. **(B)** STAT6 and EGR2-specific ChIP-seq signals in wild-type murine BMDMs after 0-, 1-, and 24-hours IL-4 stimulation, as well as RNAPII-pS2-specific ChIP-seq signals in non-polarized and IL-4-polarized wild-type (*Egr2^+/+^*) and EGR2-deficient (*Egr2^fl/fl^*) murine BMDMs at the *Vegfa* and *Flt1*-associated distal regulatory regions visualized by the Integrative Genomics Viewer. **(C)** RT-qPCR measurements of *Vegfa* mRNA expression from IL-4-polarized and non-polarized wild-type (*Egr2^+/+^*) and EGR2-deficient (*Egr2^fl/fl^*) murine BMDMs at 24 hours following IL-4 stimulation. Bar graphs present the mean ± SD of four biological replicates. **(D)** RT-qPCR measurements of *Flt1* mRNA expression from IL-4-polarized and non-polarized wild-type (*Egr2^+/+^*) and EGR2-deficient (*Egr2^fl/fl^*) murine BMDMs at 24 hours following IL-4 stimulation. Bar graphs present the mean ± SD of four biological replicates. *p <0.05, **p <0.01, ***p <0.001.

Dynamic chromatin binding of the STAT6 transcription factor was observed following the IL-4 stimulation in macrophages, showing a remarkable reduction after 24 hours of IL-4 exposure (30). In addition, it has been demonstrated that the IL-4-STAT6 signaling pathway-induced various transcription factors, including EGR2, are crucial players in the organization of the late alternative macrophage-specific epigenetic and transcriptional program (36, 56). To study whether EGR2 participates in the regulation of *Vegfa* and *Flt1* expression in the late phase of alternative macrophage polarization, we first examined the dynamics of STAT6 and EGR2 binding at their distal regulatory regions using our previously published ChIP-seq datasets (30, 36). As expected, the STAT6 binding significantly declined at the above-identified *Vegfa* and *Flt1*-associated enhancers at the late time point (24h) of alternative macrophage polarization (**Figures 4A, B**). In parallel, the EGR2 binding was dramatically induced at the previously STAT6-bound enhancers and additional distal regulatory regions in both gene loci following 24 hours of IL-4 stimulation (**Figures 4A, B**). Next, we examined the RNAPII binding at the EGR2-bound enhancers and the gene bodies in the *Vegfa* and *Flt1* loci in untreated and IL-4-stimulated (24 hours) wild-type and EGR2-deficient BMDMs using our publicly available ChIP-seq dataset (36). We found that IL-4-attenuated RNAPII binding remained detectable at the *Vegfa* gene body and its above-examined enhancers, including *Vegfa*_+278Kb and *Vegfa*_+274Kb in the EGR2-deficient macrophages, which was not observed in wild-type BMDMs (**Figures 4A, B**). In addition, three more EGR2-bound enhancers, including *Vegfa*_+225Kb, *Vegfa*_+218Kb, and *Vegfa*_+209Kb, could be identified as showing IL-4-induced and EGR2-dependent RNAPII binding (**Figures 4A, B**). In the *Flt1* locus, both the *Flt1* gene body and the EGR2-bound enhancers were associated with IL-4-induced RNAPII binding in the wild-type BMDMs, which was remarkably reduced in the absence of EGR2 (**Figures 4A, B**). To confirm the complex role of EGR2 regulating the *Vegfa-Flt1* axis at the late phase of alternative macrophage polarization, we measured their steady-state mRNA expression in unstimulated and IL-4-stimulated wild-type and EGR2-deficient BMDMs using RT-qPCR. As shown in **Figure 4C**, although IL-4 treatment was slightly inhibitory for *Vegfa* mRNA expression in wild-type BMDMs, this effect was remarkably elevated in the EGR2-deficient macrophages. Additionally, the IL-4-mediated induction of *Flt1* expression was partially attenuated in the absence of EGR2 (**Figure 4D**).

Taken together, these findings show that the EGR2 transcription factor contributes to the elimination of IL-4-dependent transcriptional repression of *Vegfa* expression and the IL-4-induced *Flt1* mRNA expression at the late phase of alternative macrophage polarization, suggesting the complex role of EGR2 in the regulation of *Vegfa* and *Flt1* expression.

### The hypoxic response of the *Vegfa*-*Flt1* axis is modified by alternative macrophage-polarizing signal IL-4

**Figure 5:**
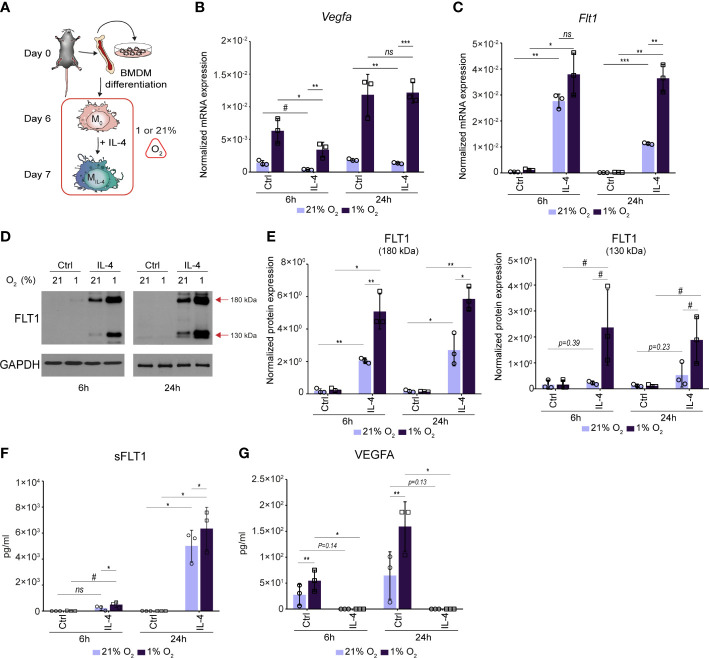
The complex interaction between IL-4 and a hypoxic environment resulting in synergistically activated FLT1 expression and completely inhibited VEGFA secretion in murine BMDMs. **(A)** Schematic representation of the mouse bone marrow isolation, BMDM differentiation and polarization by IL-4 under normoxic (21% O_2_) hypoxic (1% O_2_) conditions. **(B)** RT-qPCR measurements of *Vegfa* mRNA expression from IL-4-polarized and non-polarized wild-type BMDMs under normoxic (21% O_2_) hypoxic (1% O_2_) conditions at the indicated time points. Bar graphs present the mean ± SD of three biological replicates. **(C)** RT-qPCR measurements of *Flt1* mRNA expression from IL-4-polarized and non-polarized wild-type BMDMs under normoxic (21% O_2_) hypoxic (1% O_2_) conditions at the indicated time points. Bar graphs present the mean ± SD of three biological replicates. **(D)** Immunoblot of FLT1 expression in non-polarized and IL-4-exposed wild-type murine BMDMs under normoxic (21% O_2_) hypoxic (1% O_2_) conditions at the indicated time points. One representative blot is shown from three independent experiments. GAPDH protein expression serves as a loading control. **(E)** Immunoblot densitometry analysis of two different isoforms of FLT1 protein in IL-4-polarized and non-polarized wild-type murine BMDMs under normoxic (21% O_2_) hypoxic (1% O_2_) conditions at the indicated time points. Data represent the mean and SD of three individual animals. **(F)** ELISA measurements on the soluble FLT1 protein in culture supernatants derived from IL-4-polarized and non-polarized wild-type murine BMDMs under normoxic (21% O_2_) hypoxic (1% O_2_) conditions at the indicated time points. Bar graphs present the mean ± SD of three biological replicates. **(G)** ELISA measurements on the VEGFA protein in culture supernatants derived from IL-4-polarized and non-polarized wild-type murine BMDMs under normoxic (21% O_2_) hypoxic (1% O_2_) conditions at the indicated time points. Bar graphs present the mean ± SD of three biological replicates. #p<0.1, *p <0.05, **p <0.01, ***p <0.001, ns, not significant change.

Although macrophages can produce angiogenic factors such as VEGFA under basal conditions, various microenvironmental signals, such as hypoxia, lipopolysaccharide, nuclear receptor agonists and lactic acid can robustly enhance their VEGFA-producing capacity resulting in the development of proangiogenic macrophage phenotype (14, 15, 37, 57, 58). To investigate whether the alternative macrophage polarization can modulate the proangiogenic signals-regulated *Vegfa* and *Flt1* production, we aimed to examine the effect of IL-4 on their expression levels under hypoxic and normoxic conditions. We polarized the murine BMDMs by IL-4 in hypoxic (1% O_2_) and normoxic (21% O_2_) conditions for 6 and 24 hours and measured the *Vegfa* and *Flt1* mRNA expression using RT-qPCR (the experimental design is shown in **Figure 5A**). As expected, the hypoxia remarkably induced the *Vegfa* mRNA expression in the non-polarized BMDMs at both examined time points (**Figure 5B**). However, IL-4 had a partial antagonistic effect on the hypoxia-induced *Vegfa* expression at 6 hours after IL-4 stimulation, but this inhibitory effect was not observed later at the mRNA level (**Figure 5B**). In contrast, the hypoxic microenvironment could further enhance the IL-4-induced *Flt1* expression at both time points (**Figure 5C**). To further investigate the interaction between hypoxia and IL-4 in regulating the FLT1 and VEGFA production in macrophages, we investigated the FLT1 protein expression by immunoblot and ELISA. As shown in **Figures 5D, E**, the hypoxic microenvironment could enhance the IL-4-induced expression of both ~180 and ~130 kDa weight isoforms at the indicated time points. According to the immunoblot results, the hypoxia could further augment the IL-4-induced FLT1 secretion 6 and 24 hours after the IL-4-stimulation (**Figure 5F**). Next, we quantified the macrophage-secreted available VEGFA protein level in the cell culture media in this experimental setup by ELISA method. As expected, the hypoxia increased the VEGFA secretion at the indicated time points, but the IL-4 exposure dramatically reduced VEGFA secretion under both normoxic and hypoxic conditions (**Figure 5G**).

**Figure 6:**
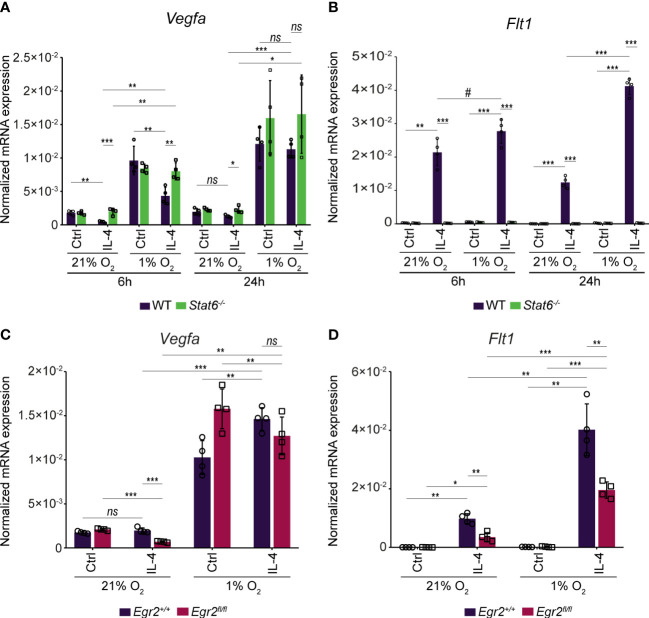
The STAT6 and EGR2 transcription factor dependency in the complex IL-4 and hypoxia-mediated regulation of *Vegfa* and *Flt1* mRNA expression in murine BMDMs. **(A)** RT-qPCR measurements of *Vegfa* mRNA expression from IL-4-polarized and non-polarized wild-type (WT) and *Stat6^-/-^* BMDMs under normoxic (21% O_2_) hypoxic (1% O_2_) conditions at the indicated time points. Bar graphs present the mean ± SD of four biological replicates. **(B)** RT-qPCR measurements of *Flt1* mRNA expression from IL-4-polarized and non-polarized wild-type (WT) and *Stat6^-/-^* BMDMs under normoxic (21% O_2_) hypoxic (1% O_2_) conditions at the indicated time points. Bar graphs present the mean ± SD of four biological replicates. **(C)** RT-qPCR measurements of *Vegfa* mRNA expression from IL-4 polarized and non-polarized wild-type (*Egr2^+/+^*) and EGR2-deficient (*Egr2^fl/fl^*) BMDMs at 24 hours after IL-4 stimulation under normoxic (21% O_2_) and hypoxic conditions (1% O_2_). Bar graphs present the mean ± SD of four biological replicates. **(D)** RT-qPCR measurements of *Flt1* mRNA expression from IL-4 polarized and non-polarized wild-type (*Egr2^+/+^*) and EGR-deficient (*Egr2^fl/fl^*) BMDMs at 24 hours after IL-4 stimulation under normoxic (21% O_2_) and hypoxic conditions (1% O_2_). Bar graphs present the mean ± SD of four biological replicates. #p<0.1, *p <0.05, **p <0.01, ***p <0.001, ns, not significant change.

Next, we wanted to study how STAT6 and EGR2 transcription factors can contribute to the IL-4-mediated transcriptional regulation of *Vegfa* and *Flt1* expression under hypoxic conditions. Therefore, we measured the *Vegfa* and *Flt1* mRNA expression in wild-type, STAT6-, and EGR2-deficient murine BMDMs in the above-described experimental conditions (**Figure 5A**). All IL-4-mediated effects on *Vegfa* and *Flt1* mRNA expression were completely abolished under normoxia and hypoxia in the absence of STAT6 (**Figures 6A, B**). In contrast to normoxia, the EGR2 deficiency could not maintain the repressive capacity of IL-4 on the *Vegfa* expression in the late phase of alternative macrophage polarization under hypoxic conditions (**Figure 6C**). Besides, the IL-4 stimulation and hypoxic microenvironment-induced synergistic activation of *Flt1* mRNA expression proved to be partially EGR2 transcription factor-dependent (**Figure 6D**).

Overall, these results demonstrate the complex interactions between the alternative macrophage polarization signal IL-4 and the hypoxic microenvironment in murine BMDMs resulting in inhibited VEGFA production and synergistically activated FLT1 expression.

### The alternative macrophage polarization-dependent regulation of the *Vegfa*-*Flt1* axis in murine tissue-resident macrophage subsets

**Figure 7:**
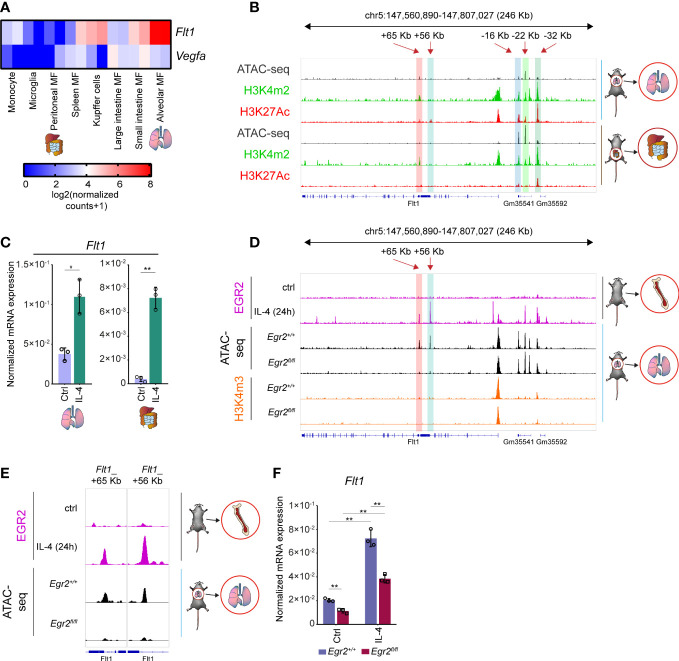
The transcriptional regulation of basal and IL-4-induced *Flt1* expression in murine alveolar and peritoneal macrophages. **(A)** Heatmap representation of the basal *Flt1* and *Vegfa* mRNA expression in murine monocytes and seven different tissue-resident macrophage subsets (n=2). **(B)** H3K4m2 and H3K27Ac-specific ChIP-seq and ATAC-seq signals in wild-type murine alveolar and peritoneal macrophages at the *Flt1* locus visualized by the Integrative Genomics Viewer. **(C)** RT-qPCR measurements of *Flt1* mRNA expression from IL-4 polarized and non-polarized wild-type alveolar and peritoneal macrophages at 6 hours after IL-4 stimulation. Bar graphs present the mean ± SD of three biological replicates. **(D)** EGR2-specific ChIP-seq signals in wild-type IL-4 polarized and non-polarized BMDMs, as well as H3K4m3-specific CUT&RUN-sequencing and ATAC-seq signals in wild-type (*Egr2^+/+^*) and EGR2-deficient (*Egr2^fl/fl^*) alveolar macrophages at the *Flt1* locus visualized by the Integrative Genomics Viewer. **(E)** EGR2-specific ChIP-seq signals in wild-type IL-4 polarized and non-polarized BMDMs, as well as an ATAC-seq signals in wild-type (*Egr2^+/+^*) and EGR2-deficient (*Egr2^fl/fl^*) alveolar macrophages at two intronic enhancers of *Flt1* gene visualized by the Integrative Genomics Viewer. **(F)** RT-qPCR measurements of *Flt1* mRNA expression from IL-4 polarized and non-polarized wild-type (*Egr2^+/+^*) and EGR2-deficient (*Egr2^fl/fl^*) alveolar macrophages at 6 hours after IL-4 stimulation. Bar graphs present the mean ± SD of three biological replicates. *p <0.05, **p <0.01.

It is well known that the distinct tissue-resident macrophage populations differ from the bone marrow and blood monocyte-derived macrophages, including their origin and epigenetic program (6, 59). Therefore, we decided to examine whether the IL-4-dependent regulation of *Vegfa* and *Flt1* mRNA expression is restricted to the BMDMs or is more widely observable in various murine tissue-resident macrophage subsets. To do this, we investigated the basal *Vegfa* and *Flt1* mRNA expression in monocytes and seven tissue-resident macrophage populations by re-analyzing a publicly available RNA-seq dataset (59). As shown in **Figure 7A**, *Vegfa* was expressed at a relatively low level in each macrophage subtype, while the *Flt1* expression showed remarkable differences between the different tissue-resident macrophage subsets. The most abundant *Flt1* expression was detected in the alveolar macrophages and the Kupffer cells, and the small intestine macrophages also expressed it at a relatively high level (**Figure 7A**). In contrast, the basal *Flt1* mRNA expression level was low in the peritoneal macrophages and the microglial cells (**Figure 7A**). To study the epigenetic background of the distinct basal mRNA expression pattern of *Flt1*, we selected two tissue-resident macrophage populations from the endpoints of the *Flt1* expression scale, including alveolar and peritoneal macrophages. We investigated the chromatin accessibility, as well as H3K4m2 and H3K27Ac post-translational histone modification patterns in the *Flt1* locus re-analyzing publicly available ATAC-seq and ChIP-seq datasets (59). Based on the epigenetic marks, we could identify many distal regulatory regions from the above-described IL-4-responsible genomic sites, including *Flt1*_+65 Kb, *Flt1*_+56 Kb, *Flt1*_-16 Kb, *Flt1*_-22 Kb, and *Flt1*_-32 Kb enhancers, in both macrophage subtypes (**Figure 7B**). We did not find remarkable differences in the chromatin openness and H3Km2 pattern at the *Flt1*-associated enhancers and its promoter when comparing the alveolar and peritoneal macrophages (**Figure 7B**). However, the active transcription mark H3K27Ac was significantly elevated at the promoter and the enhancers in the alveolar macrophages explaining the different basal *Flt1* mRNA expression levels between the peritoneal and alveolar macrophages (**Figure 7B**).

In order to determine whether IL-4 can modulate the basal *Flt1* and *Vegfa* expression levels in the selected tissue-resident macrophages, we isolated alveolar and peritoneal macrophages with FACS sorting, stimulated them by IL-4 for 6 hours and measured the mRNA expression using RT-qPCR. As expected, the basal *Flt1* expression proved to be higher in alveolar macrophages, but IL-4 could significantly enhance it in both cell types (**Figure 7C**). Additionally, the basal *Vegfa* mRNA level was also reduced in the examined macrophage subpopulations by IL-4, similar to the BMDMs (**Supplementary Figure 3A**).

It has been previously described that EGR2 plays an important role not just in the late phase of alternative macrophage polarization, but it is also expressed at a high level in alveolar macrophages and contributes to their proper maturation (36, 56, 60). Therefore, we wanted to investigate whether EGR2 can influence the basal *Flt1* mRNA level and the IL-4-dependent regulation of both *Vegfa* and *Flt1* expression in the alveolar macrophages using ATAC-seq, ChIP-seq, and RT-qPCR methods. First, we examined the chromatin accessibility in the *Flt1* locus at those regions, which were bound by EGR2 in the alternatively polarized BMDMs. Among these genomic regions, we could identify two intronic enhancers, including *Flt1*_+65 Kb and *Flt1*_+56 Kb, showing dramatically reduced chromatin openness in EGR2-deficient alveolar macrophages compared to the wild-type counterparts (**Figures 7D, E**). Additionally, the active promoter mark H3K4m3 enrichment was reduced around the transcription start site of the *Flt1* gene in the absence of EGR2 in alveolar macrophages, raising the possibility that EGR2 is important for the proper expression of *Flt1* in murine alveolar macrophages (**Figure 7D**). To confirm this hypothesis, we measured the *Flt1* expression in non-polarized and alternatively polarized wild-type and EGR2-deficient murine alveolar macrophages by RT-qPCR. As expected, both the basal and IL-4-induced *Flt1* mRNA level was significantly diminished in the absence of EGR2 (**Figure 7F**). Since the EGR2 was also involved in regulating IL-4-mediated *Vegfa* repression in murine BMDM, we also examined this phenomenon in the EGR2-deficient alveolar macrophages. In contrast to the BMDM, the EGR2 deficiency resulted in higher *Vegfa* mRNA expression in the IL-4-stimulated alveolar macrophages compared to the wild-type alternatively polarized alveolar macrophages (**Supplementary Figure 3B**).

Taken together, these findings suggest that the complex alternative macrophage polarization-dependent regulation of the *Vegfa* and *Flt1* expression also exists in different murine tissue-resident macrophage populations.

### The expression pattern of the *Vegfa*-*Flt1* axis *in vivo* in different parasite infection models

**Figure 8:**
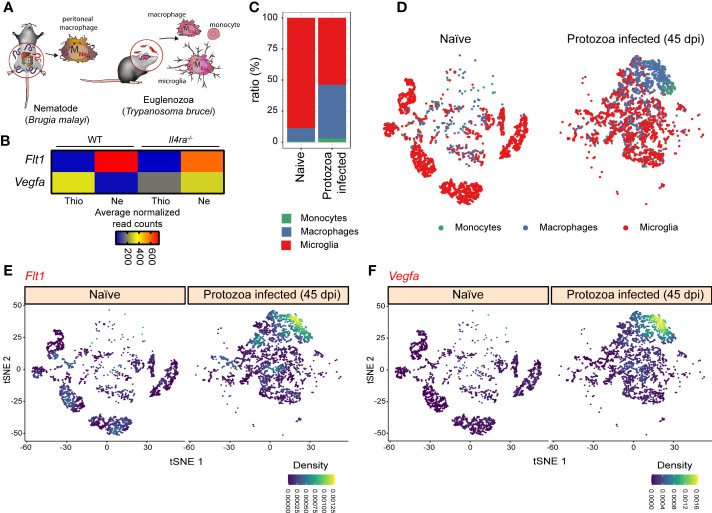
The expression pattern of *Vegfa-Flt1* axis in the alternatively polarized macrophages derived from two different *in vivo* parasite infection models. **(A)** Schematic representation of the *in vivo* alternative macrophage polarization inducing filarial nematode *Brugia malayi* and protozoa parasite *Trypanosoma brucei* infection models. **(B)** Heatmap representation of the mRNA expression of *Flt1* and *Vegfa* in thioglycolate (Thio) and *Brugia malayi* nematode-elicited (Ne) wild-type (WT) and IL4Rα-deficient (*Il4ra^-/-^*) peritoneal macrophages (n=3). **(C)** The proportion of the monocytes, macrophages, and microglial cells in the hypothalamic regions derived from naïve and *Trypanosoma brucei*-infected mice. **(D)** tSNE plot of hypothalamic monocytes, macrophages, and microglial cells derived from naïve and *Trypanosoma brucei*-infected mice. **(E)** tSNE plot shows the *Flt1* mRNA expression in hypothalamic monocytes, macrophages, and microglial cells derived from naïve and *Trypanosoma brucei*-infected mice. **(F)** tSNE plot shows the *Vegfa* mRNA expression in hypothalamic monocytes, macrophages, and microglial cells derived from naïve and *Trypanosoma brucei*-infected mice.

Next, we wanted to study whether the alternative macrophage polarization-specific regulation of the *Flt1*-*Vegfa* axis is observable *in vivo.* For this, we selected a bulk RNA-seq dataset derived from the filarial nematode *Brugia malayi* (Model I.) and a single-cell RNA-seq dataset derived from the protozoan parasite *Trypanosoma brucei* (Model II.) infection models because both the *Brugia malayi* infection and the late phase of *Trypanosoma brucei* infection are associated with alternative macrophage polarization (the scheme of parasite infections is shown in **Figure 8A**) (61–64). First, we determined the *Vegfa* and *Flt1* mRNA expression in *Brugia malayi*-elicited alternatively polarized peritoneal macrophages, which expressed the well-known alternative macrophage polarization markers at a high level (**Supplementary Figure 4A**). According to our *in vitro* and *ex vivo* observations, the *Flt1* expression was significantly induced, while *Vegfa* mRNA level was attenuated in the nematode-elicited alternatively polarized peritoneal macrophages compared to the thioglycolate-elicited macrophages (**Figure 8B**). To determine whether the nematode infection-regulated *Vegfa* and *Flt1* mRNA expression are IL-4 dependent, we investigated their expression levels in thioglycolate and nematode-elicited peritoneal macrophages derived from *Il4ra^-/-^* mice. Similar to the induction of well-known alternative macrophage polarization markers, the repression of *Vegfa* expression was completely abolished in the absence of IL4Rα (**Figure 8B** and **Supplementary Figure 4A**). Additionally, the nematode infection-induced *Flt1* mRNA level also showed a partial IL4Rα dependency (**Figure 8B**). Next, we investigated the *Vegfa* and *Flt1* mRNA expression levels in the hypothalamic monocytes, macrophages and microglial cells derived from naïve and *Trypanosoma brucei-*infected mice 45 days after the parasite infection. As shown in **Figures 8C, D**, and **Supplementary Figures 4B−D**, both the number of macrophages and the mRNA levels of *Chil3* and *Arg1* in this macrophage population were remarkably elevated in the hypothalamus derived from *Trypanosoma brucei-*infected mice. According to the elevated expression levels of alternative macrophage polarization markers, both *Flt1* and *Vegfa* genes were expressed robustly in the hypothalamic macrophages at the late stage of *Trypanosoma brucei* infection (**Figures 8E, F** and **Supplementary Figure 4D**).

Overall, these results indicate that *Flt1* was induced in the alternatively polarized macrophages during different parasite infections, while the *in vivo* regulation of the *Vegfa* expression depends on the parasite infection models. Nevertheless, the determination of the functional consequences of this modified macrophage-expressed *Vegfa* and *Flt1* pattern in parasite infections requires further experimental work.

### IL-4 modulates VEGFA and FLT1 expression in human differentiating macrophages under normoxic and hypoxic conditions

**Figure 9:**
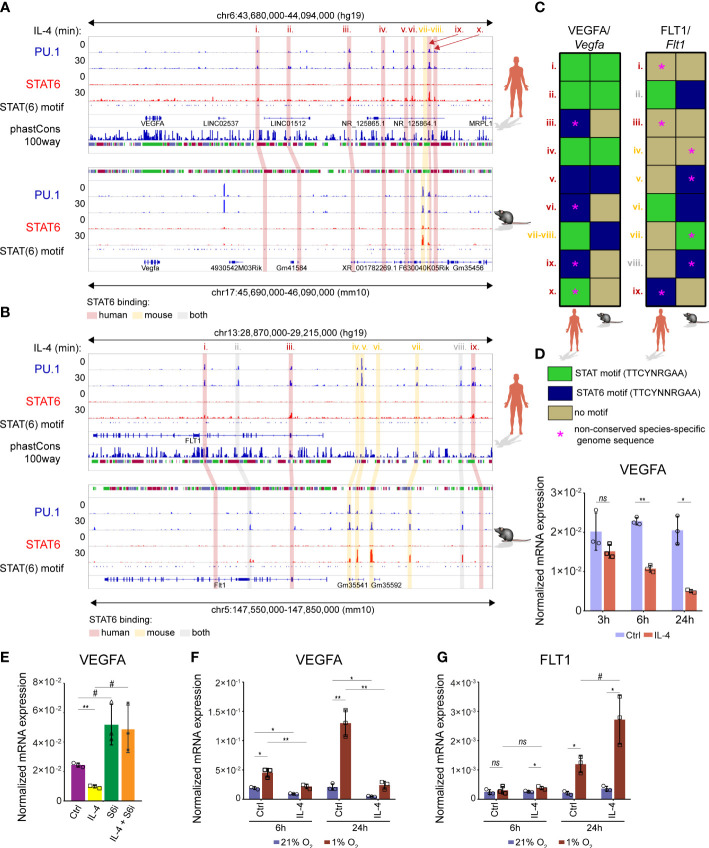
Similarities and differences in the IL-4-dependent transcriptional regulation of VEGFA and FLT1 expression between murine and human macrophages. **(A)** PU.1 and STAT6-specific ChIP-seq signals in IL-4-polarized and non-polarized wild-type murine BMDMs and human differentiating macrophages at the *Vegfa*/VEGFA loci visualized by the Integrative Genomics Viewer. Conservation scores of the VEGFA locus are presented in the phastCons track, followed by the track that shows the presence of STAT(6) binding motifs. **(B)** PU.1 and STAT6-specific ChIP-seq signals in IL-4-polarized and non-polarized wild-type murine BMDMs and human differentiating macrophages at the *Flt1*/FLT1 loci visualized by the Integrative Genomics Viewer. Conservation scores of the VEGFA locus are presented in the phastCons track, followed by the track that shows the presence of STAT(6) binding motifs. **(C)** Heatmap showing the presence of canonical STAT and STAT6-specific motifs at PU.1 and STAT6 co-bound enhancers in mouse and human *Vegfa*/VEGFA and *Flt1*/FLT1 genomic loci. **(D)** RT-qPCR measurements of VEGFA mRNA expression from IL-4 polarized and non-polarized human CD14^+^ monocyte-derived differentiating macrophages at the indicated time points. Bar graphs present the mean ± SD of three biological replicates. **(E)** RT-qPCR measurements of VEGFA mRNA expression from IL-4 polarized and non-polarized human CD14^+^ monocyte-derived differentiating macrophages in the absence and presence of STAT6 phosphorylation inhibitor (S6i; 1 µM) at 6 hours after the IL-4 stimulation. Bar graphs present the mean ± SD of three biological replicates. **(F)** RT-qPCR measurements of VEGFA mRNA expression from IL-4 polarized and non-polarized human CD14^+^ monocyte-derived differentiating macrophages at the indicated time points, under normoxic (21% O_2_) and hypoxic conditions (1% O_2_). Bar graphs present the mean ± SD of three biological replicates. **(G)** RT-qPCR measurements of FLT1 mRNA expression from IL-4 polarized and non-polarized human CD14^+^ monocyte-derived differentiating macrophages at the indicated time points, under normoxic (21% O_2_) and hypoxic conditions (1% O_2_). Bar graphs present the mean ± SD of three biological replicates. #p<0.1, *p <0.05, **p <0.01, ns, not significant change.

Although the members of the IL-4-activated signaling pathway are evolutionarily conserved between the mouse and human macrophages, a remarkable divergence is also observable in the alternative macrophage polarization-specific transcriptional programs (22, 65). To study whether the IL-4-dependent regulation of the VEGFA-FLT1 axis is evolutionarily conserved, we determined the genome sequence conservation at both examined gene loci and investigated the lineage-determining transcription factor PU.1 and STAT6 binding patterns in non-polarized and IL-4-activated murine BMDMs and human CD14 positive monocyte-derived differentiating macrophages using our previously published ChIP-seq datasets (30, 38). As the phastCons tracks show in **Figures 9A, B**, remarkable sequence conservation was observed in both gene loci between humans and mice. Additionally, similarly to the murine macrophages, IL-4-activated STAT6 binding was identified in the PU.1 positive FLT1 and VEGFA-associated distal regulatory regions in human differentiating macrophages (**Figures 9A, B**). Despite these facts, the IL-4-activated STAT6 binding only slightly overlapped between the human and murine alternatively polarized macrophages (**Figures 9A, B**). Additionally, the sequence conservation under the STAT6 peaks, and the presence of the general STAT and STAT6-specific elements, showed remarkable differences between the examined species (**Figures 9A−C**). To investigate whether the distinct STAT6 binding pattern results in different IL-4 responsiveness between humans and mice, we measured the VEGFA and FLT1 expression patterns in IL-4-polarized human monocyte-derived differentiating macrophages at different time points using RT-qPCR. As shown in **Figure 9D**, IL-4 could repress VEGFA expression at the 6 and 24 hour time points. In contrast, FLT1 was not regulated at any time points by IL-4 (data not shown). Next, we examined the STAT6 dependency of IL-4-mediated VEGFA repression using the specific STAT6 inhibitor AS1517499. As expected, the pharmacological inhibition of STAT6 could diminish the inhibitory effect of IL-4 on VEGFA mRNA expression (**Figure 9E**). Finally, we studied the VEGFA and FLT1 mRNA expression in alternatively polarized human differentiating macrophages under hypoxic conditions. Similarly to the murine BMDMs, IL-4 could partially repress the hypoxia-induced VEGFA expression at both examined time points (**Figure 9F**). Intriguingly, the long-term (24 hours) hypoxia could significantly induce the FLT1 mRNA level, and IL-4 could further enhance it in the human macrophages (**Figure 9G**).

These findings indicate that the IL-4-dependent regulation of VEGFA and FLT1 expression show partial similarities under normoxic and hypoxic conditions between murine and human macrophages despite the observed negligible overlap in the STAT6-bound regulatory regions.

## Discussion

Understanding macrophage participation in regulating physiological and pathological angiogenesis is essential and can contribute to developing new therapeutic strategies to target the different stages of pathological vessel formation in various diseases and tissue engineering to repair and regenerate complex tissues and organs functionally. Although our current knowledge about the angiogenesis-regulating role of differently polarized macrophages is quite controversial, a growing number of evidence indicates that the alternatively polarized or anti-inflammatory macrophages contribute to the later phases of angiogenesis, including sprouting and vessel remodeling or regression (21, 27). These macrophage states are often associated with a specific expression pattern of the angiogenesis-linked genes including the reduced level of proangiogenic VEGFA and elevated production of the VEGFR family member FLT1 with antiangiogenic capacity (17, 21, 29). However, the regulatory mechanisms leading to this angiogenesis-modulating program in these macrophage subtypes are not completely understood.

Our current study uncovered hitherto unknown regulatory mechanisms controlling the VEGFA production in different alternatively polarized murine and human macrophages. On the one hand, we could confirm the previous observations about the IL-4-inhibited VEGFA production in macrophages (21, 66). On the other hand, we demonstrated that the IL-4-STAT6 signaling pathway could efficiently reduce the *Vegfa*/VEGFA mRNA expression in murine and human macrophages with species and/or macrophage subtype-specific differences. In murine BMDMs and human monocyte-derived differentiating macrophages, direct STAT6 binding at the *Vegfa*/VEGFA associated enhancers and complete STAT6 dependency of the repression indicate the direct transcriptional repressor activity of STAT6, confirming our previous observations about IL-4-STAT6 signaling pathway-attenuated gene expression (30). However, remarkable differences could be detected in the duration of IL-4-dependent repression of *Vegfa*/VEGFA expression between human and mouse macrophages. The IL-4-induced EGR2 transcription factor was able to suspend the transcriptional inhibitory effects of the STAT6 on *Vegfa* expression, resulting in the almost completely eliminated repression 24 hours after IL-4 exposure in the mouse macrophages. Although the EGR2 is also induced in human macrophages (36), the IL-4-STAT6 signaling pathway-mediated repression of VEGFA mRNA level proved to be the most pronounced at the late phase of alternative macrophage polarization. We assume that the reason for these species-specific differences is that although the phenomenon of IL-4-STAT6-mediated repression is observed in both species, the STAT6-bound enhancers differ in murine and human macrophages. Nevertheless, verifying the distinct EGR2 binding pattern at the STAT6-bound enhancers annotated to the VEGFA gene requires further experimental confirmation.

Regarding the alternative macrophage polarization-specific regulation of antiangiogenic *Flt1* expression, we investigated transcriptional and epigenetic bases of the IL-4 inducibility of different *Flt1* isoforms at mRNA and protein levels in murine and human macrophages. Similar to previous publications (29, 67, 68), we could also demonstrate the IL-4-dependent induction of *Flt1* in various bone marrow-derived and tissue-resident murine macrophages. The Flt1 locus contains previously identified “early sustained” and “late” IL-4-activated enhancers (36). The STAT6 binding at the “early sustained” enhancers is responsible for the early direct activation of *Flt1* transcription, while the IL-4-induced EGR2 binding at the “early sustained” and “late” enhancers contributes to the robust *Flt1* expression in the late phase of alternative macrophage polarization. Interestingly, EGR2, as one of the key transcription factors in the alveolar macrophages (36, 60), also provides the high basal level and IL-4 inducibility of *Flt1* expression in alveolar macrophages. Although the FLT1-inducible effect of IL-4 was negligible in human monocyte-derived macrophages under normoxic conditions, it could further enhance the hypoxic microenvironment-induced FLT1 expression after 24 hours of co-treatment. These findings raised the possibility that the STAT6 or the IL-4-STAT6 signaling pathway-induced additional transcription factors can facilitate the responsiveness to various microenvironmental changes in a gene-specific manner, similar to the liganded RXR-facilitated IL-4 response of CCL26 and IL1RN genes in the human differentiating macrophages (40).

In general, an important feature of immunomodulatory cytokines is that they can modify the macrophage response to various external and internal signals or the changing microenvironment. The altered responsiveness is often caused by epigenetic and transcriptional regulatory mechanisms. Perhaps the best-characterized examples of the altered macrophage responsiveness are the modified TLR activation-induced inflammatory program in the IFNγ or IL-4-primed macrophages (30, 69–72). However, local hypoxia also occurs frequently in the microenvironment of macrophages in various physiological or pathological processes, significantly enhancing the proangiogenic capacity of macrophages through the elevated production of VEGFA and any other proangiogenic factors (14, 73, 74). Some pieces of evidence indicate that various immunomodulatory cytokines in the microenvironment can significantly modify the angiogenic and VEGFA-producing capacities of the local myeloid compartment. Among others, TGFβ enhances the *Vegfa* expression in murine macrophages and dendritic cells (75, 76). In contrast, IL-10 has a microenvironment-specific regulatory role of *Vegfa* expression in murine BMDMs by attenuating its hypoxia-induced expression in the presence of IFNγ (77). Here we observed that IL-4 can specifically modulate the hypoxic response to the *Vegfa*/VEGFA and *Flt1*/FLT1 genes in both murine and human macrophages. Similarly to the normoxic condition, the IL-4-STAT6 signaling pathway reduced the *Vegfa*/VEGFA expression and the available biologically active VEGFA level in the macrophage cell culture supernatant. Besides, IL-4 and hypoxia could synergistically induce *Flt1*/FLT1 expression in both murine BMDMs and human monocyte-derived differentiating macrophages. These findings illustrate that the cytokine microenvironment remarkably influences the hypoxia-activate proangiogenic program in macrophages. Furthermore, the modulatory role of alternative macrophage polarization signal IL-4 is not restricted to the TLR activation-mediated inflammatory response (30, 69), but it also influences the expression of the hypoxia-activated angiogenesis-associated genes in both murine and human macrophages.

Despite the long-accepted VEGFA antagonizing effect of sFLT1 (78, 79), the potential role of alternatively polarized macrophage-produced FLT1 is not completely understood. On the one hand, it was demonstrated that the alternatively polarized macrophage-derived factors, including sFLT1, could efficiently block the vessel formation *in vitro*, and recombinant IL-4 administration attenuated the laser-induced choroidal neovascularization *in vivo* through the infiltrating macrophages-derived sFLT1 (29). On the other hand, other studies revealed that the alternatively polarized macrophages have proangiogenic capacity *in vitro* and *in vivo* (25, 26). Thirdly, alternative macrophage polarization is often associated with pathological processes characterized by increased angiogenesis including tumor development and metastasis formation, parasite infections, or Th2-type airway inflammation and asthma (24, 80–83). For instance, an important adaptation strategy of filarial parasites can be the induction of blood vessel formation following the infection (84). Indeed, *Heligmosomoides polygyrus* infection enhanced the levels of several proangiogenic factors in cerebrospinal fluid and lead to new vessel formation in the brain (83). Using publicly available bulk RNA-seq and single-cell RNA-seq datasets from two different parasite infection models, we determined that the nematode infection-induced alternative macrophage polarization was linked to elevated *Flt1* mRNA level and repressed *Vegfa* expression. In contrast, the protozoa infection-induced alternative macrophage polarization and *Flt1* expression were associated with robust *Vegfa* mRNA expression. These findings indicate that parasite infection-specific differences are observed in the regulation of angiogenic gene signature in the alternatively polarized macrophages. Nevertheless, these findings raised the possibility that the alternatively polarized macrophage-produced FLT1 can contribute to the defense response of the host against the parasite infection through VEGFA neutralization and angiogenesis inhibition. However, currently there is no direct experimental evidence for this theory, and it seems more likely that the alternatively polarized macrophage-expressed FLT1 does not exclusively regulate angiogenesis *in vivo* in different pathological conditions. Accordingly, FLT1 is induced in the metastasis-associated macrophages at both mRNA and cell surface protein levels in an IL-4-dependent manner (68). It has been demonstrated that the macrophage-expressed FLT1 is critical for metastases formation regulating a set of inflammatory genes and colony-stimulating factor 1 (85). Identifying those factors or circumstances influencing the dominance of the antiangiogenic effect of sFLT1 or the signal pathway mediated by the membrane-bound FLT1 requires further investigation under different pathological conditions.

Overall, our work here provides insight into how IL-4 can regulate *Vegfa*/VEGFA and *Flt1*/FLT1 expression at the transcriptional level in different murine and human macrophage subtypes *in vitro* and *ex vivo*. The described STAT6-mediated direct repression of *Vegfa*/VEGFA expression is observed under normoxic and hypoxic conditions but showed species-specific characteristics. In parallel with this, the IL-4-STAT6-EGR2 regulatory axis-mediated enhancement of *Flt1* expression is only detected in murine macrophages under normoxia. However, IL-4 and hypoxia can synergistically induce *Flt1*/FLT1 expression in murine and human macrophages. Finally, the *in vivo* parasite infection generally leads to elevated *Flt1* expression in the alternatively polarized murine macrophages derived from the peritoneal cavity and the brain, while the regulation of *Vegfa* mRNA level depends on the site and/or species of parasite infection.

## Data availability statement

The original contributions presented in the study are included in the article/**Supplementary Material**. Further inquiries can be directed to the corresponding author.

## Ethics statement

The studies involving human participants were reviewed and approved by Peripheral blood was collected from healthy volunteers from the Regional Blood Center of the Hungarian National Blood Transfusion Service (Debrecen, Hungary) with the approval of the Regional Institutional Research Ethics Committee of the University of Debrecen. Written informed consent for participation was not required for this study in accordance with the national legislation and the institutional requirements. The animal study was reviewed and approved by the Animal Care and Protection Committee at the University of Debrecen (16/2019/DE MAB).

## Author contributions

Conception and design: ZC. Generate experimental data: AD; ZV; KJ; ES; KB; AM. Analysis and interpretation of data: AD; ZV; KJ; ES; NC-S; GN; BS; LH; EV; ZC. Writing of the manuscript: AD; ZV and ZC. Study supervision: GS; AB; VJ; LN and ZC. All authors contributed to the article and approved the submitted version.
