# Supplementary figures and images for: The transcriptional control of the VEGFA-VEGFR1 (FLT1) axis in alternatively polarized murine and human macrophages
Source: Front Immunol. 2023 May 4;14:1168635. doi: 10.3389/fimmu.2023.1168635 (PMC10192733; doi:10.3389/fimmu.2023.1168635)

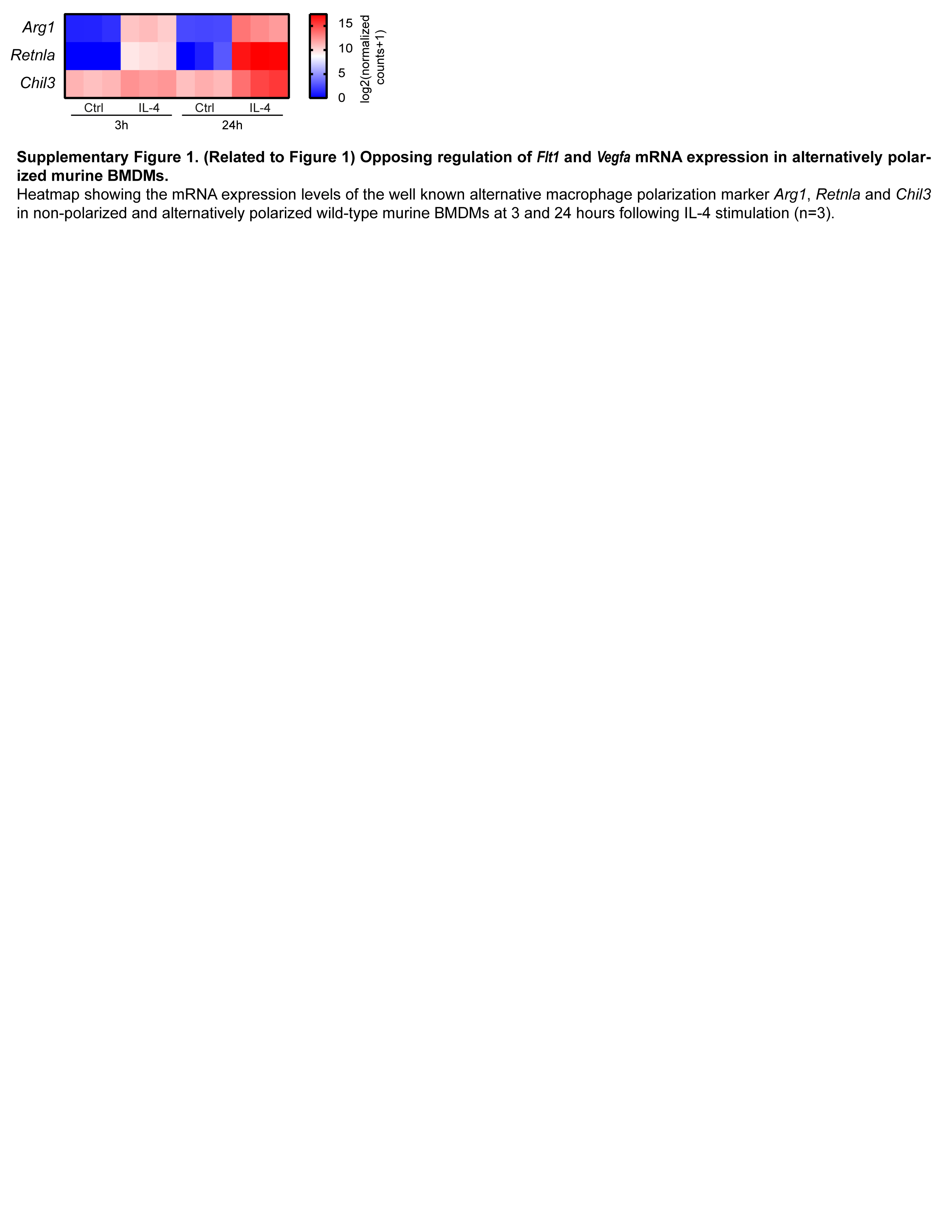

Supplement: Supplementary file 1 [file Image_1.tif]

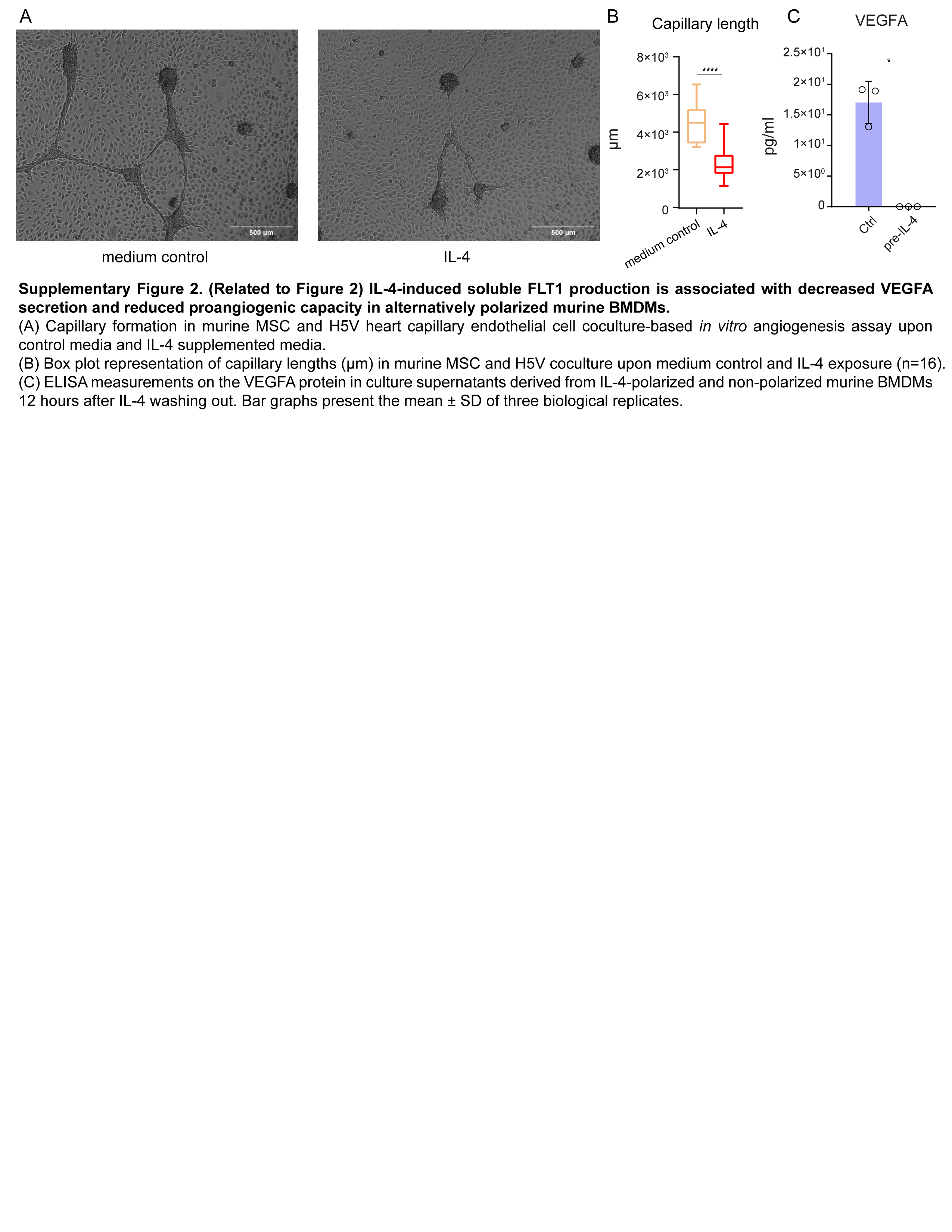

Supplement: Supplementary file 2 [file Image_2.tif]

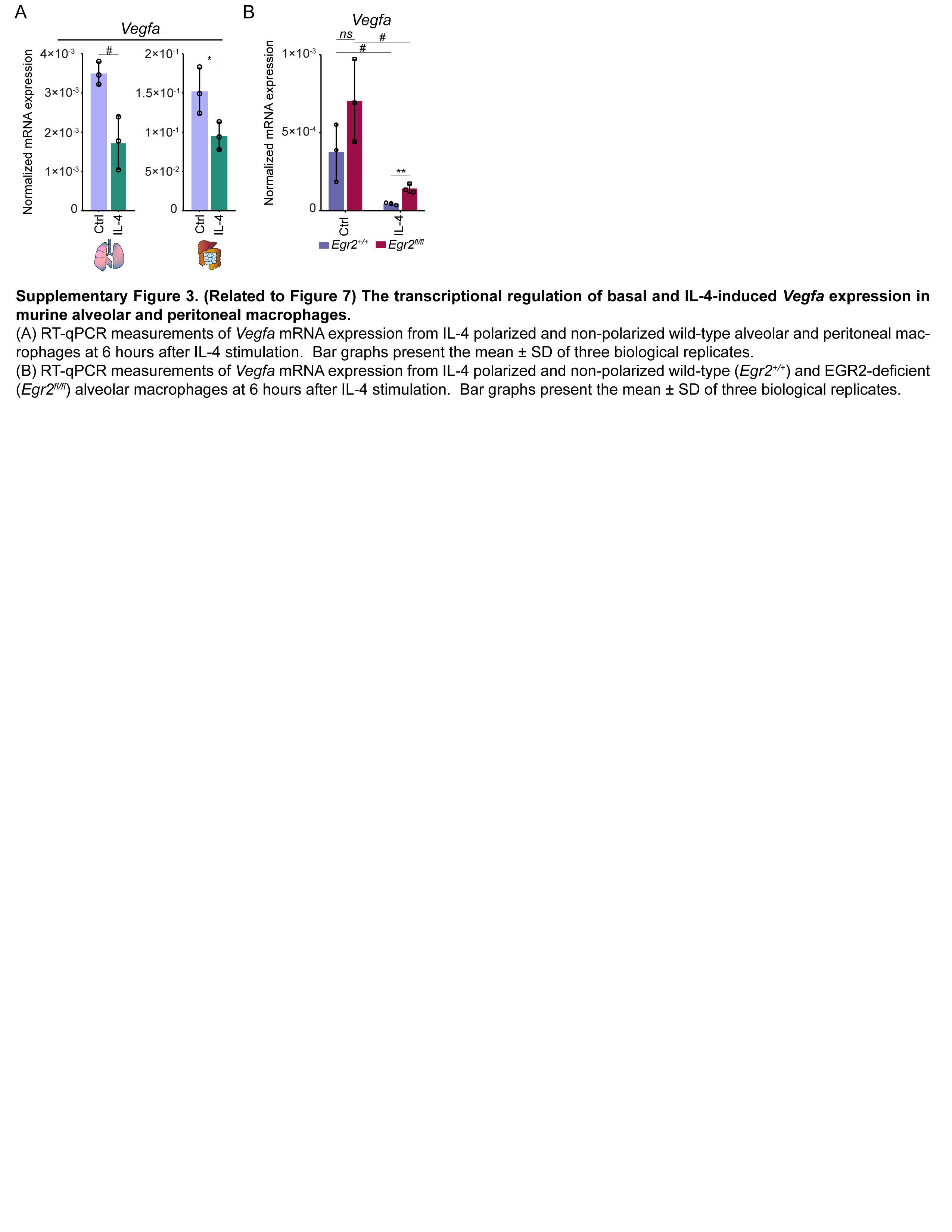

Supplement: Supplementary file 3 [file Image_3.tif]

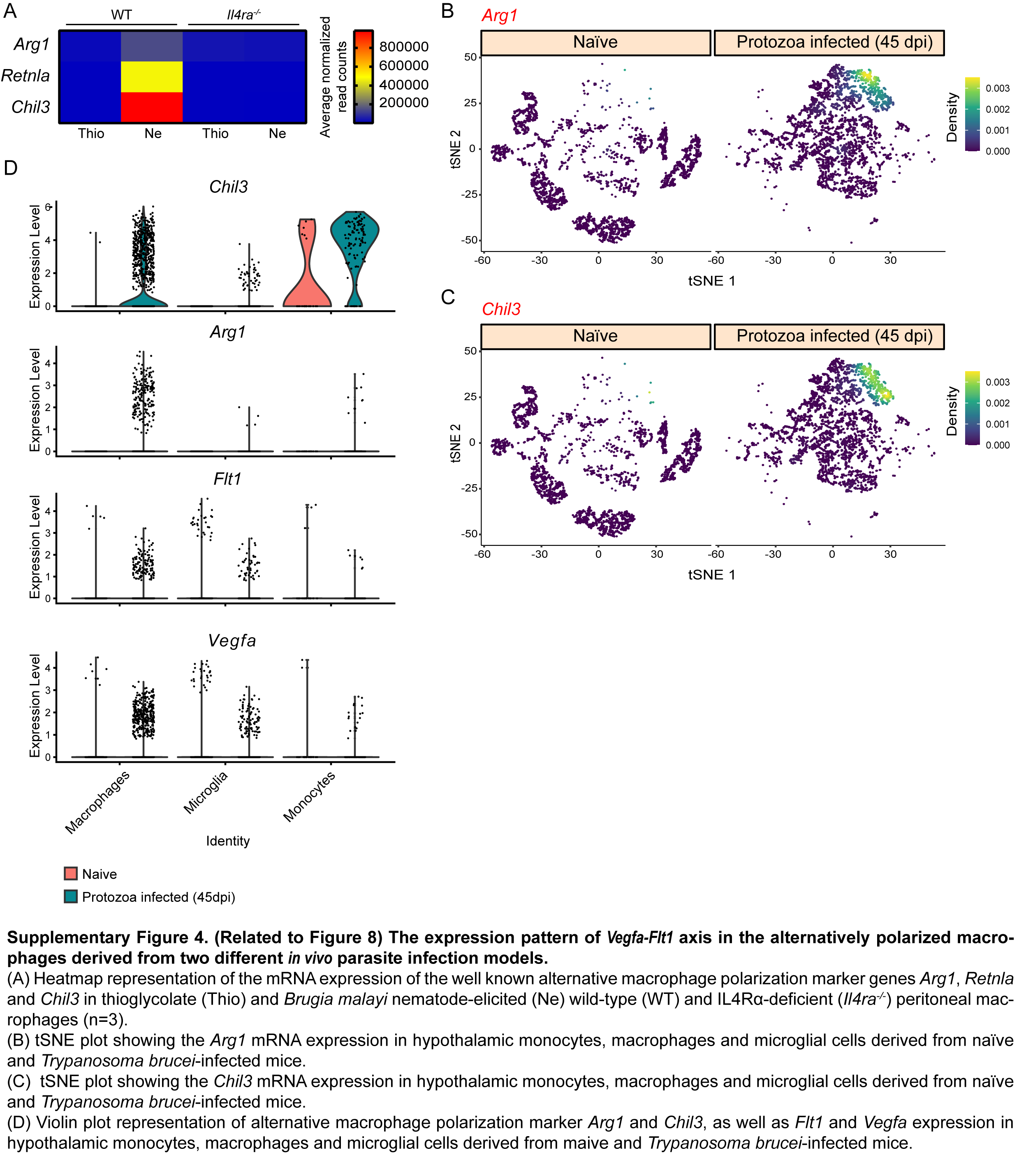

Supplement: Supplementary file 4 [file Image_4.tif]
